# Green Chemistry Meets Olive Mill Wastewater: Bioinspired Oxidation of Phenols and Polyphenols Using Selenium Catalysts

**DOI:** 10.3390/ijms26115192

**Published:** 2025-05-28

**Authors:** Cecilia Scimmi, Izabela Szymanek, Diana Rogacz, Sebastiano Passeri, Giulia Patanella, Cezary Kozłowski, Małgorzata Deska, Piotr Rychter, Jozef Drabowicz, Claudio Santi

**Affiliations:** 1 Group of Catalysis, Sinthesis and Organic Green Chemistry, Department of Pharmacuetical Sciences, University of Perugia, Via del Liceo 1, 06123 Perugia, Italy; cecilia.scimmi@dottorandi.unipg.it (C.S.); sebastianopasseri1@gmail.com (S.P.); giulia.patanella@studenti.unipg.it (G.P.); 2 Faculty of Science and Technology, Jan Długosz University in Częstochowa, 13/15 Armii Krajowej Av., 42-200 Częstochowa, Poland; izabela.szymanek@doktorant.ujd.edu.pl (I.S.); d.rogacz@ujd.edu.pl (D.R.); c.kozlowski@ujd.edu.pl (C.K.); m.deska@ujd.edu.pl (M.D.); p.rychter@ujd.edu.pl (P.R.); j.drabowicz@ujd.edu.pl (J.D.); 3 Division of Organic Chemistry, Center of Molecular and Macromolecular Studies, Polish Academy of Sciences, 112 Sienkiewicza, 90-363 Lodz, Poland

**Keywords:** selenium, catalysis, bioinspired, toxicology, *Aliivibrio fischeri*, Microtox, olive mill wastewater, circular economy

## Abstract

Olive mill wastewater (OMW) represents a toxic waste generated during olive oil production (30 million m^3^/year). Its phytotoxicity and resistance to biodegradation are mainly due to the presence of polyphenols. Methodologies able to remove these organic compounds from this waste to allow the safe dispose of OMW have been developed, and among them, the most effective are oxidation procedures. In this context, we propose an alternative chemical treatment based on the oxidation of OMW using diluted hydrogen peroxide and seleno-organic compounds (diphenyl diselenide and diseleno-bis-benzoic acid) selected as eco-friendly bioinspired catalysts. The effectiveness of the protocol was monitored by Folin–Ciocalteu (F-C) quantification and NMR quantification. The results demonstrated that the greatest reduction in the total phenols content—up to 96%—was achieved using the highest concentrations of catalyst (0.6% *_w_*_/*w*_) and oxidant (10% *_v_*_/*v*_). Moreover, a toxicological evaluation was carried out using the marine bacteria *Aliivibrio fischeri*, revealing a significant decrease in toxicity. The EC_50_ value increased from 0.089 mg/L in the untreated OMW to 18.740 mg/L in the treated sample after removal of the residual catalyst and peroxides.

## 1. Introduction

Olive oil is a functional food of extreme importance in the Mediterranean diet [1]. Its annual production fluctuates between 2.5 and 3 million of tons: the International Council of Oil reports that in the 2022–2023 season, 2.56 million tons were generated. The leading countries in this sector are Spain (400,000 tons per year), followed by Italy (250,000–400,000 tons per year) and all the other Mediterranean countries (40,000–250,000 tons per year), which together supply 98% of the worldwide olive oil demand [2,3]. One of the most abundant wastes that derives from olive oil extraction is olive mill wastewater (OMW) [2,3]. Representing an aqueous biomass produced in an estimated amount of 30 million m^3^ per year [4], the term OMW indicates all the water generated during olive oil production. The composition of OMW is really variable, but generally, the main components are the liquid fraction (83–94%), suspended particles (kernel, olive peel, olive pulp), organic components (4–16%) and inorganic compounds (0.4–2.5%) [5]. This waste, characterized by a very dark brown color and unpleasant smell, is known as a toxic effluent, and specifically, the pollution generated from 1 m^3^ of OMW is equivalent to that produced by 100–200 m^3^ of domestic sewage [6]. The toxicity is due to different factors, such as the low pH (3–5), the very high level of phosphorus salts [7] and the high chemical and biochemical oxygen demand (COD and BOD) of up to 100 and 200 g/L [8]. Among all the organic constituents, the most toxic phenols, accounting for 98%, are concentrated in OMW and just 2% remain in the oil during the extraction process [9]. Considered the principal cause of OMW’s toxicity, they are responsible for the phytotoxicity of OMW, inhibiting the seeds’ growth and germination, and for the resistance to biodegradation, especially against anaerobic microorganisms [8,10]. What are described above are the main reasons why the disposal of OMW is a problem, especially for all the countries that produced olive oil in huge amounts. Indeed, OMW cannot be directly discharged into the sewer [7,11] and direct dispose on fields is forbidden, unless correctly performed (e.g., for Italy, law L. 574/96 describes all the procedures to use for the disposal on fields in terms of the amount of OMW/ha, period of disposal, type of land). The common procedure to dispose of OMW involves using artificial ponds, but this does not represent a resolution of the problem. Indeed, the organic compounds (e.g., polyphenols) are concentrated during evaporation, making OMW even more toxic, the ponds are not waterproof, and the highly polluting waste leaks into the nearby fields and aquifers [12]. Another aspect to take into consideration is the bothersome odors generated, especially during the hot months of the year, due to the presence of low-molecular-weight carboxylic acids (butyric, caproic, valeric acid [7]).

For this reason, different treatments aiming to lower OMW’s toxicity and allow the safe and not environmentally dangerous dispose of this waste were developed. Considering that the main components responsible for OMW’s toxicity are polyphenols, all the processes share the common goal of removing these organic compounds from OMW and thus obtaining a very precious biomass. Indeed, taking into consideration the water scarcity problem that afflicts the world [13], the detoxified OMW can be recycled and used to irrigate and fertilize fields thanks to its very high nitrogen content [14]. The most common procedures applied can be discriminated into physical, biological and chemical procedures. In the physical treatment of OMW, the removal of polyphenols is combined with their recyclability; indeed, given their anticancer, antioxidant and cardioprotective properties, these compounds can be retrieved and reused in pharmaceutical formulations [15]. One of the primary challenges associated with the recovery of phenols is the difficulty of releasing them. As is often the case with absorption procedures, while approximately 90% of polyphenols are effectively removed from OMW and retained on the absorbent materials, the remaining 10% or so are not fully released, resulting in the recovery of less than 50% of the initial polyphenol content [16]. This ultimately renders the recyclability of the absorbent material unfeasible [17]. Moreover, to be effective, the physical procedures have to be applied in combination with other treatments; for example, ultrafiltration can be followed by reverse osmosis [18], or coagulation can be combined with electrooxidation [19].

On the contrary, both biological and chemical treatments aim to achieve the polyphenols’ elimination through a degradation process. *Azotobacter vinelandii* [20], *Bacillus pumilus* [21], *Pseudomonas and Ralstonia* [22], *Pseudomonas putida* DSM 1868 and *Ralstonia* sp. LD35 are some examples of aerobic bacteria applied in OMW’s biological treatment. To this class also belongs anaerobic digestion in which the polyphenol content reduction is followed by methanogenesis [8].

The most effective chemical treatments for OMW polyphenols’ degradation are advanced oxidative processes (AOPs) and wet air oxidation (WAO). The latter are carried out using high pressure (2–20 MPa) and high temperature [23] and can be performed under non-catalyzed conditions, as in the work of Mantzavinos [23], or, and this is the most common procedure, in the presence of a heterogenous recoverable catalyst such as Ir, Pt or CuO supported on carbon (Ir/C and Pt/C) [24,25] or supported on Al_2_O_3_ [25]. Differently, AOPs consist of oxidative treatments performed at ambient temperature and pressure. During these processes, the formation of radicals (e.g., OH^•^, H_2_O_2_^•^, O_2_^•−^, O_3_^•^, HO_3_^•−^, HO_2_^•^) that derive from oxidants such as H_2_O_2_, O_2_ and O_3_ is essential to convert the substrates into more degradable products, obtaining as results the mineralization of organic compounds into H_2_O and CO_2_ [26]. In the case of OMW, depending on the sources of the energy used to promote the production of radicals, AOPs are grouped in photooxidation [27,28], sono-oxidation [29], electrooxidation [19] and Fenton and Fenton-like reactions. Among all of them, the last type of reaction is the most used and consists of the oxidation of polyphenols using H_2_O_2_ and catalyzed by iron containing salts, both homogenous (e.g., Fe(OH)_3_) [30] and heterogenous [31].

Given the high toxicity and environmental impact of OMW, particularly due to its high concentrations of phenolic compounds, lipids, and reduced carbohydrates, it is essential to monitor and evaluate its ecotoxicological effects before and after treatment. Traditional chemical analyses, while informative, often fail to capture the complex biological interactions and cumulative toxicity of such heterogeneous mixtures. Tests involving living organisms are widely used in environmental, pharmaceutical, and industrial research to assess the toxicity of various chemical compounds. These methods enable the evaluation of a substance’s impact on organisms at different biological levels, from the cellular to ecosystem scale. Their application allows for monitoring potential risks to human and animal health, as well as the natural environment, and assessing the effectiveness of wastewater and industrial waste treatment processes. Among the most used organisms for assessing the toxicity of chemical substances are the marine bacteria *Aliivibrio fischeri* and freshwater crustaceans such as *Heterocypris incongruens* or *Daphnia magna*. These organisms serve as biological models in ecotoxicological tests due to their high sensitivity to pollutants and the ease of conducting laboratory studies. Polyphenols, commonly found in OMW, are known to have significant toxicity to bacteria such as *Aliivibrio fischeri*. These compounds can inhibit bioluminescence, a measure of bacterial activity, indicating their harmful impact on microbial life. The toxicity is often attributed to the phenolic compounds’ ability to disrupt cellular functions. As such, *A. fischeri* is often used as a bioindicator to assess the environmental toxicity of polyphenol-rich substances like OMW, helping to understand their potential ecological impact [12,13,14,15,16,17,18,19,20,21,22,23,24,25,26,27,28,29,30,31,32]. Therefore, bioassays like the Microtox test, which utilizes these bacteria, offer a rapid, sensitive, and reliable method to assess the toxicity of OMW and its treated derivatives. These bioassays provide valuable insight into the potential ecological risks associated with OMW disposal and can be effectively integrated into wastewater treatment evaluation protocols to ensure environmental safety and compliance with regulatory standards. An example of such an approach is the research conducted by Gotsi et al. [33] and Mekki et al. [34], who examined the impact of OMW on these organisms. Their experiments provided valuable insights into the toxicity levels of OMW and its potential effects on aquatic ecosystems. A similar methodology for assessing toxicity was applied in the study by Belaqziz et al. [35], where the impact of olive mill wastewater on microbial communities present in the soil filter was investigated using an infiltration–percolation system as a treatment method. The aquatic bacteria *A. fischeri* were used to evaluate the effectiveness of this approach in reducing the harmful environmental impact of these wastewaters. Considering the toxicity levels used to evaluate the response of *A. fischeri* to various substances, Hernando et al. [36] proposed the following classification: substances with an EC_50_ < 1 mg/L are classified as “very toxic to aquatic organisms”, those with an EC_50_ between 1 and 10 mg/L as “toxic”, and those with an EC_50_ ranging from 10 to 100 mg/L as “harmful”. Given the demonstrated ecotoxicity of polyphenol-rich wastewaters such as OMW and the relevance of using *A. fischeri* as a bioindicator to assess their impact, it becomes imperative to explore environmentally benign treatment strategies that can effectively reduce their toxicity.

In this context, we explore an alternative and eco-friendly oxidative catalytic protocol, selecting as the biomimetic catalyst an organoselenium compound. Selenium derivatives are known to be oxygen transfer catalysts from the oxidant reagent to the substrate, bioinspired by the activity of glutathione peroxidase (GPx) in promoting the oxidation of numerous organic substrates [37]. They are considered eco-friendly catalysts because they can be used in combination with green oxidants such as hydrogen peroxide, without the need for harsh conditions or high pressure and temperature, are compatible with green solvents such as ionic liquids and water, even if insoluble in this reaction media, and their recyclability has been demonstrated [38]. This new procedure is cheaper than physical methods and more easily applicable than biological treatments. Furthermore, it can be considered more environmentally friendly with respect to other oxidative methods, considering that it can be carried out without the use of extreme conditions such as high temperatures and pressures, as reported for WAO [23].

## 2. Results and Discussion

### 2.1. Oxidation of Model Compounds

**Figure 1:**
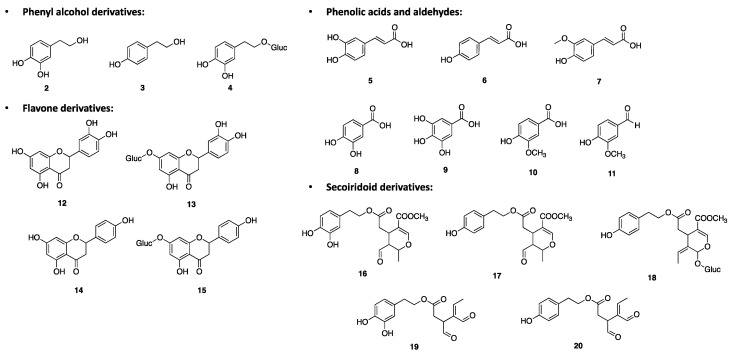
Polyphenols in OMW.

In this work, H_2_O_2_ (30%) was chosen as an environmentally friendly oxidant, while the cheap and commercially available diphenyl diselenide (PhSe)_2_ (**1**)) was tested as a catalyst. The investigation started with a few model compounds selected as mimics of the most representative derivatives among more than 50 different phenolic compounds identified and classified as phenyl alcohol derivatives (**2**–**4**), phenolic acids and aldehydes (**5**–**11**), flavonoids (**14**–**15**) and secoiridoid derivatives (**16**–**20**) (Figure 1) [9].

Catechol (**21**) was chosen as a representative of the alcoholic aromatic portion of hydroxytirosol (**3**) and oleuropein (**16**), two of the most abundant polyphenols in OMW. To mimic the aqueous condition of OMW, the reactions were carried out in water using 10 mol% equivalents of **1** and 30 mol. equivalents of the oxidant. Through monitoring the reactions by ^1^H-NMR (see Supplementary Materials Figure S1), it was possible to identify a quantitative conversion of **21** after 30 min, obtaining as the main product the Z,Z-muconic acid (**22**), in the presence of maleic acid **23a** and fumaric acid **23b** in lower amounts (Entry 1, Table 1).

**Scheme 1:**
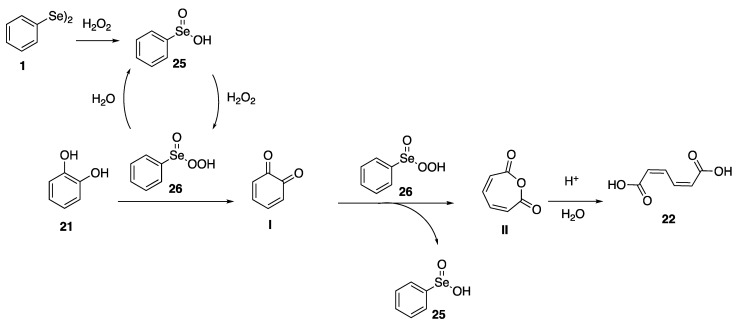
Proposed mechanism for the oxidation of **21** [34].

As reported by Giurg et al. [39], the formation of **22** occurs through the oxidative ring opening of **21** after its initial conversion into the orto-benzoquinone (**I**). The intermediate **I** undergoes an intramolecular Baeyer–Villiger reaction and the subsequent hydrolysis produces the dicarboxylic acid **22**. As can be seen from the scheme below, the actual catalyst capable of promoting both the oxidation of **21** and the Baeyer–Villiger reaction is phenyl perseleninic acid (**26**), obtained by the oxidation of **1** (Scheme 1).

**Scheme 2:**
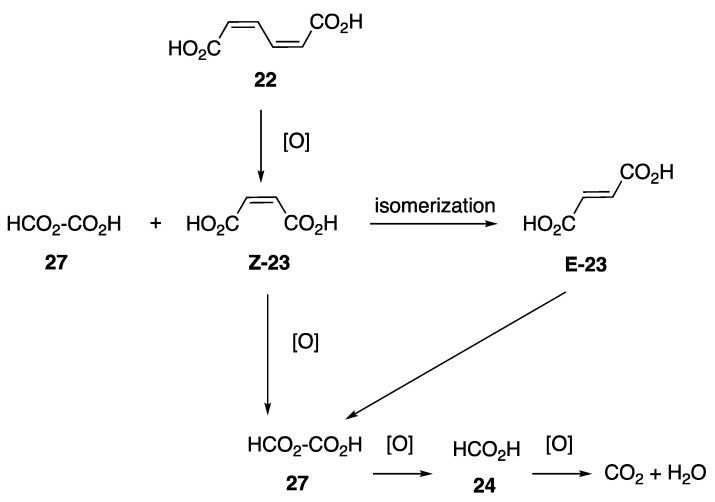
Degradation of **22**.

Surprisingly, running the reaction for an additional half an hour (Entry 2, Table 1) resulted in a significant decrease in the NMR yield (from 99% to 65%), while the conversion was quantitative, as in Entry 1. In addition, a lower percentage of **22** was observed as well an increase in the production of **E-23**, **Z-23** and **24**. This peculiar trend is even more noticeable when the reaction time is increased to 24 h. In fact, while **21** is completely converted into the products, the NMR yield reaches the very poor value of 17%, and as observed before, the equilibrium is shifted toward the formation of **23** and **24** rather than **22** (Entry 5, Table 1). We hypothesized that this phenomenon is due to the degradation of compound **22**, leading to a loss of mass of the crude, despite the 100% conversion of **21**. Indeed, **22** in the presence of an excess of oxidant can be converted into maleic acid **Z-23**, which is oxidized directly or after isomerization into **E-23** to oxalic acid **27** and formic acid **24** [40]. Finally, **24** can be further oxidized into H_2_O and CO_2_, which was detected through a CO_2_ trap (Scheme 2).

The optimized oxidation conditions were then applied to other phenolic substrates, some with the free hydroxyl functions, others protected as methyl esters (**3**, **28**–**33**). The results presented in Table 2 show that, with the exception of substrate **3** (Supplementary Materials Figure S4), all the substrates were successfully converted into oxidation products in 30 min. In particular, as observed for catechol, phenol (**28**) is quantitatively oxidized to give **22** as the main product (Supplementary Materials Figure S2), while in the case of **29**, the conversion reaches the value of 80% and the most abundant products are the corresponding muconic acid **34** and muconolactone **35**, obtained by an intramolecular Michael reaction of **34** [39]. From the ^1^H-NMR spectrum of the crude reaction mixture, products **36** and **32** were also identified, representing two intermediates in the oxidation of **29** (Supplementary Materials Figure S3). In the case of compound **30**, representative of the resorcinol part of flavones, and **31** and **33**, representative of the aromatic part of some phenolic acids and aldehydes in OMW, the reaction resulted in the complete oxidation of the substrates, with **Z-23** being identified as the main product in all cases (Supplementary Materials Figures S5, S6 and S8). Also, for compound **32**, **34** and **35** are the main products obtained with 100% conversion, while **E-23** and **24** are trace products (Supplementary Materials Figure S7).

### 2.2. Measurement of Polyphenol Amount by F-C Method

The next step was the application of the oxidative protocol to the raw material, aiming to reduce the concentration of polyphenols responsible for the toxicity of OMW [41]. Three different conditions in terms of the catalyst and oxidant amount were also investigated in the presence of three different sources of light: UV (395–405 nm), Blue LED (465 nm) and Green LED (495 nm). The effectiveness of this protocol was evaluated by means of the Folin–Ciocalteu (F-C) quantification of the polyphenols.

**Figure 2:**
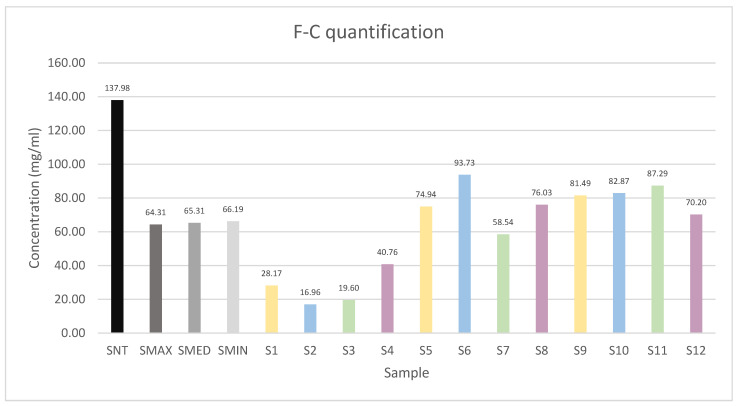
F-C quantification of OMW samples. **SNT:** OMW non-treated; **SMAX:** OMW treated with H_2_O_2_ (10% *_v_*_/*v*_); **SMED:** OMW treated with H_2_O_2_ (1% *_v_*_/*v*_); **SMIN:** OMW treated with H_2_O_2_ (0.1% *_v_*_/*v*_); **S1:** OMW treated with H_2_O_2_ (10% *_v_*_/*v*_) + (PhSe)_2_ (0.6% *_w_*_/*w*_) at room condition; **S2:** OMW treated with H_2_O_2_ (10% *_v_*_/*v*_) + (PhSe)_2_ (0.6% *_w_*_/*w*_) under Blue LED irradiation; **S3:** OMW treated with H_2_O_2_ (10% *_v_*_/*v*_) + (PhSe)_2_ (0.6% *_w_*_/*w*_) under Green LED irradiation; **S4:** OMW treated with H_2_O_2_ (10% *_v_*_/*v*_) + (PhSe)_2_ (0.6% _W/W_) under UV irradiation; **S5:** OMW treated with H_2_O_2_ (1% *_v_*_/*v*_) + (PhSe)_2_ (0.06% *_w_*_/*w*_) at room condition; **S6:** OMW treated with H_2_O_2_ (1% *_v_*_/*v*_) + (PhSe)_2_ (0.06% *_w_*_/*w*_) under Blue LED irradiation; **S7:** OMW treated with H_2_O_2_ (1% *_v_*_/*v*_) + (PhSe)_2_ (0.06% *_w_*_/*w*_) under Green LED irradiation; **S8:** OMW treated with H_2_O_2_ (1% *_v_*_/*v*_) + (PhSe)_2_ (0.06% *_w_*_/*w*_) under UV irradiation; **S9:** OMW treated with H_2_O_2_ (0.1% *_v_*_/*v*_) + (PhSe)_2_ (0.006% *_w_*_/*w*_) under room conditions; **S10:** OMW treated with H_2_O_2_ (0.1% *_v_*_/*v*_) + (PhSe)_2_ (0.006% *_w_*_/*w*_) under Blue LED irradiation; **S11:** OMW treated with H_2_O_2_ (0.1% *_v_*_/*v*_) + (PhSe)_2_ (0.006% *_w_*_/*w*_) under Green LED irradiation; **S12:** OMW treated with H_2_O_2_ (0.1% *_v_*_/*v*_) + (PhSe)_2_ (0.006% *_w_*_/*w*_) under UV irradiation.

The graph reported in Figure 2 shows the results of these quantifications in a series of differently treated OMW samples (see Supplementary Materials Table S1 and Figure S9). The highest TPs (total phenols) concentration was detected for the untreated OMW (**SNT**: 137.98 mg/mL), while for all the other oxidized samples, the TPs concentration is lower, confirming that the applied oxidative protocol produces the phenols’ and polyphenols’ degradation, as was predictable by the preliminarily results obtained on the model compounds.

The best results were obtained when the highest amounts of the catalyst (PhSe)_2_ **1** and oxidant (H_2_O_2_) were used, [0.6% _(*w*/*w*)_ and 10% _(*v*/*v*)_, respectively] affording, after 24 h under room conditions (**S1**), a TPs concentration of 28.17 mg/mL, corresponding to an 80% reduction in the total initial amount. The role of the catalyst was demonstrated by performing the reaction with only H_2_O_2_ at the maximum amount (**SMAX**), obtaining a moderate reduction (54%) in TPs similar compared to that observed using medium (**SMED**) and minimum (**SMIN**) amounts of oxidant (65.31 mg/mL and 66.19 mg/mL, respectively). When the treatment was associated with different light irradiation, Blue LED **S2** and Green LED **S3** led to the highest reduction in the TPs concentration, while UV irradiation **S4** seemed to be less effective. Using the lowest amount of the catalyst and oxidant, the effectiveness of the protocol is strongly reduced and apparently non-selenium-catalyzed, affording comparable results to the reaction performed without the catalyst (**SMED** and **SMIN**). One of the main drawbacks of the direct application of the F-C assay on a matrix such as OMW consists of the overestimation of the TPs concentration due to the presence of other reducing species [42]. In fact, reducing sugars, some proteins and amino acids, as well as vitamin C, can interfere in the redox reaction that occurs between the phenols and the F-C reagent, resulting in an overestimation of the real TPs concentration [42,43]. For this reason, the F-C assay was applied not only directly on OMW but also on the samples extracted with EtOAc [44]. The results reported in Figure S10a and Table S2 (see Supplementary Materials) show that even if the detected TPs concentration is significantly lower, the overall trend of the effects produced by the different treatments is unchanged. Additional ^1^H-NMR experiments were performed to explain why, with this latest protocol, the oxidation carried out using lower amounts of the catalyst and oxidant [(PhSe)_2_ (0.006% *_w_*_/*w*_)_—_H_2_O_2_ (0.1% *_v_*_/*v*_)] leads to a greater decrease in the total phenols content compared to the intermediate conditions [(PhSe)_2_ (0.06% *_w_*_/*w*_) and H_2_O_2_ (1% *_v_*_/*v*_)]. The analysis of the aqueous phase after the extraction demonstrated that polyphenols are still present in the water treated with intermediate (**S5**) and minimum (**S9**) amounts of the catalyst and oxidant (Supplementary Materials Figure S11). A similar behavior was observed even when the extraction was carried out in acidic conditions (pH = 2) [45], a protocol that is generally considered the most effective procedure for quantitative TPs content determination (Supplementary Materials Figure S12a and Table S3).

### 2.3. Measurement of Polyphenol Amount by qNMR

These results demonstrated that the F-C quantification is non-compatible with our oxidative protocol, and for this reason, we decided to use qNMR techniques for the polyphenols quantification directly on the treated OMW, avoiding the extraction step. The results obtained after treatment of the OMW with the maximum [(PhSe)_2_ (0.6% *_w_*_/*w*_)_—_H_2_O_2_ (10% *_v_*_/*v*_)] medium [(PhSe)_2_ (0.06% *_w_*_/*w*_)_—_H_2_O_2_ (1% *_v_*_/*v*_)] and low [(PhSe)_2_ (0.006% *_w_*_/*w*_)_—_H_2_O_2_ (0.1% *_v_*_/*v*_)] amounts of the catalyst/oxidant are summarized in Table 3, Table 4 and Table 5, respectively.

From the ^1^H-NMR analysis of the chemical shifts characteristic of polyphenols, it was possible to identify and quantify compound **37**, the methoxylated form of oleocanthal, tyrosol (**3**) and hydroxytirosol (**2**) (Supplementary Materials Figure S13a). A TPs concentration of 4.70 mg/mL was detected for the untreated OMW (**SNT**, Table 3) as a mixture of 0.45 mg/mL of **3**, 2.91 mg/mL of **2** and 1.35 mg/mL of **37**. Oxidation in H_2_O_2_ (10% _(*v*/*v*)_) without the catalyst afforded a TPs content decrease of 49% (**SMAX**, Table 3). The use of 0.6% _(*w*/*w*)_ diphenyl diselenide as a catalyst resulted in a degradation of TPs ranging from 92 to 96% across the various conditions (**S1**–**S4**, Table 3). In the given conditions, compound **2** was fully consumed, while **3** and **37** were strongly reduced. No appreciable differences were observed among the different sources of irradiation used.

A lower amount of H_2_O_2_ and a lower amount of catalyst resulted in the reduced effectiveness of the polyphenols’ degradative oxidation. The results reported in Table 4 and Table 5 show that medium and low amounts of the oxidant/catalyst produce only a moderate TPs content reduction, ranging from 33 to 47% and 32 to 38%, respectively (see Supplementary Materials Figure S13b,c).

### 2.4. Toxicological Evaluation

With this evidence in hand, a toxicological evaluation of the OMW samples was carried out using the *Aliivibrio fischeri* test, a spectroscopic analysis based on the bioluminescence of bacteria.

The changes in the EC_50_ values are presented in Table 6.

**Figure 3:**
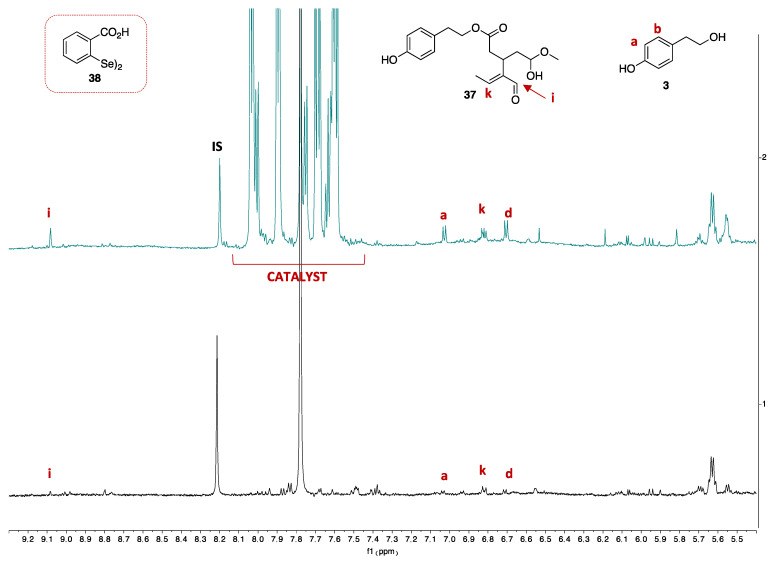
Spectrum of OMW treated with DSBA (green); and spectrum of OMW treated with DSBA after treatment with AmberLite^®^ IRA-900 and silica (black).

The analysis of the EC_50_ values obtained on *A. fischeri* bacteria indicates that the highest toxicity is observed in sample **SNT** (untreated OMW). In the case of samples **S1**–**S4** (treated with H_2_O_2_ 10% and (PhSe)_2_ 0.6%), although they show a decrease in toxicity compared to **SNT**, they still remain relatively toxic. A comparable EC_50_ value was observed for samples **S1***–***S2**, even if the ^1^H-NMR quantification showed the highest reduction in the TPs content. On the other hand, a reduction in the concentration of H_2_O_2_ and (PhSe)_2_ combined with irradiation leads to a further decrease in OMW’s toxicity, as observed in samples **S5**–**S8** (H_2_O_2_ 1% and (PhSe)_2_ 0.06%). Samples **S9***–***S12** (H_2_O_2_ 0.1% and (PhSe)_2_ 0.006%) had the lowest concentration of H_2_O_2_ and (PhSe)_2_, reflecting the lowest toxicity against the examined bacteria, particularly in sample **S11** (irradiated with Green LED). The EC_50_ values of these samples are significantly higher, indicating very low toxicity compared to the initial samples. All the used treatments decreased the toxicity of OMW, but according to Hernando’s classification, the samples still are considered “very toxic to aquatic organisms”) [37]. The presence of the actual catalyst and the unreacted peroxides detected in samples **S1**–**S4** at a level of 10 mg/mL were hypothesized to explain this peculiar trend (Supplementary Materials Figure S13a). Based on the ^77^Se-NMR analysis, the selenium derivative present in the treated OMW as a new contaminant is benzenselenonic acid (**38**) with a chemical shift of 1024.9 ppm [46] (Supplementary Materials Figure S14). Despite several efforts, we were not able to remove the catalyst from the water. For this reason, we decided to investigate as the catalyst diselenobis benzoic acid (DSBA (**38**), Figure 3) because the presence of a free carboxylic acid should enable its removal by treatment with a basic resin in heterogeneous conditions.

After treatment of the OMW for 24 h using DSBA **38** (0.6% *_w_*_/*w*_) and H_2_O_2_ (10% *_v_*_/*v*_) under room conditions, the NMR quantification evidenced an 87% reduction in the TPs content. As evidenced by the NMR spectra reported in Figure 3, the catalyst was successfully removed by fluxing the oxidized sample through a column packed with AmberLite^®^ IRA-900 and silica. Furthermore, the spectra show also that phenols **3** and **37** are still present in the sample after AmberLite^®^ treatment (Figure 3, see also Supplementary Materials Figures S15 and S16).

**Figure 4:**
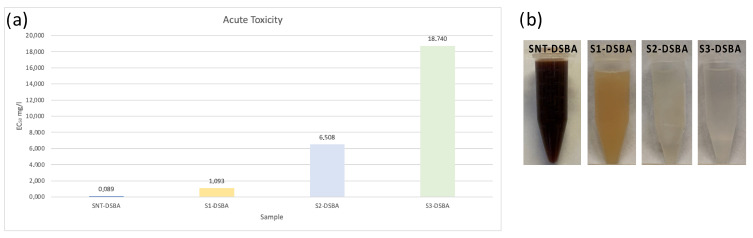
(**a**) Microtox^®^ EC_50_ values (mg/L) of the exposure of the luminescent marine bacteria *A. fischeri* to the tested OMW samples after 5 min; and (**b**) picture of the OMW samples. **SNT-DSBA:** OMW non-treated; **S1-DSBA:** OMW treated with H_2_O_2_ (10% *_v_*_/*v*_) + DSBA (0.6% *_w_*_/*w*_) under room conditions; **S2-DSBA:** OMW after catalyst removal; and **S3-DSBA:** OMW after catalyst removal and UV irradiation.

The EC_50_ values (Table 7 and Figure 4) illustrate the changes in the toxicity of the OMW samples after different treatment stages. The untreated sample (**SNT**) has the lowest EC_50_ value (0.089 mg/L), reflecting relatively high toxicity. After treatment with hydrogen peroxide (H_2_O_2_) coupled with **DSBA**, the EC_50_ value increases to 1.093 mg/L (**S1-DSBA**), suggesting that this type of chemical treatment reduces the sample’s toxicity. After catalyst removal (**S2-DSBA**), the EC_50_ rises almost ten times to 6.508 mg/L, indicating an additional decrease in toxicity. The highest EC_50_ value (18.74 mg/L) is observed in sample **S3-DSBA**, which underwent both catalyst removal and UV irradiation treatment, suggesting the most effective reduction in toxicity. The systematic increase in the EC_50_ values across the samples demonstrates that each treatment stage progressively decreases the toxicity, with the combination of catalyst removal and UV exposure showing the most significant effect. The higher the EC_50_ value, the greater the concentration required to achieve the same biological effect, confirming a substantial reduction in toxicity or activity after the applied purification methods.

Analysis of the literature points out that phenolic compounds generally demonstrate a harmful effect on *A. fischeri* (Babić et al. [47]). They proved the high toxicity of the non-treated OMW (EC_50_ = 0.24 mg/L); however, after chemical modification of the polar fraction, the EC_50_ value slightly increased to 0.67 mg/L. In our study, the value of the non-treated OMW was even lower (0.089 mg/L), indicating higher toxicity. This slight difference may result from the differing chemical compositions of wastewater from various sources, including variations in the phenolic content and other organic substances. They examined also the crustacea *Daphnia magna* (EC_50_ = 1.43 mg/L), algae *Chlorella vulgaris* (EC_50_ = 5.20 mg/L), and zebrafish *Danio rerio* (EC_50_ = 7.05 mg/L). However, it was emphasized that *A. fischeri* bacteria show the highest sensitivity to such substances, making them an effective model for assessing OMW toxicity. In the study by Isidori et al. [48], the EC_50_ value of non-treated OMW for *A. fischeri* was 0.268 mmol/L, which corresponds to approximately 0.024 mg/L, indicating even greater toxicity compared to our results. This clearly emphasizes the importance of effectively treating such compounds. It also highlights the need for a thorough assessment of the potential toxicity of complex matrices, such as OMW, on various organisms within the food chain, particularly water species, which may be especially vulnerable to the impact of OMW runoff. The EC_50_ values of the non-treated OMW, both in our study and in other reports, are generally classified as “very toxic to aquatic organisms” according to Hernando et al. [37]. However, effective purification methods have led to a significant reduction in polyphenols’ toxicity impact.

## 3. Materials and Methods

### 3.1. General

The solvent reagents and commercially available starting materials were purchased from Sigma-Aldrich (St. Louis, MO, USA), Alfa Aesar (Kandel, Germany), and VWR (Milano, Italy), and they were used as received unless otherwise noted. The reactions were performed in round-bottom flasks or Schlenk flasks and were stirred with Teflon-coated magnetic stirring bars. The analytical thin-layer chromatography (TLC) was performed on Merck silica gel 60 F254 (Merck, Darmstadt, Germany) precoated aluminum foil sheets and visualized by UV irradiation or by iodine staining. Sigma Aldrich silica gel (230–400 mesh) was used for the flash chromatography and silica gel Kieselgel 60 (70–230 mesh) was used for the column chromatography. The quantification experiments were performed using a UV/Vis spectrophotometer (Tecan infinite 200Pro, in a Greiner 96/384 flat bottom transparent polystyrene, Tecan Austria GmbH, Grodig, Austria). The centrifugation was performed using the centrifuge NF 800R (Nuve, Ankara, Turkey). The light photoreaction setup was realized using the procedure reported in [49]. The olive mill wastewater was collected during the season 2021 by EcoTech Engineering s.p.a. from an olive oil producer located in Gualdo Cattaneo, Umbria Region, Italy, and stored at 0 °C. The NMR experiments were performed at 25 °C on a Bruker Avance Neo (Bruker, Fällanden, Switzerland) spectrometer operating at 400 MHz with a sample case or spectrometer operating at 600 MHz with a CryoProbe Prodigy and sample jet, for ^1^H and 50.31 or 100.62 MHz for ^13^C. The ^1^H and ^13^C chemical shifts (δ) are reported in parts per million (ppm) and they are relative to TMS 0.0 ppm and the residual solvent peak of CDCl_3_ at δ 7.26 and δ 77.00 in the ^1^H and ^13^C NMR, respectively. The data are reported as follows: chemical shift (multiplicity, number of hydrogens, coupling constants where applicable, and assignment where possible). The abbreviations are as follows: s (singlet), d (doublet), t (triplet), q (quartet), dd (doublet of doublet), dt (double of triplet), tt (triplet of triplet), m (multiplet), br s (broad signal). The coupling constant (J) is quoted in Hertz (Hz) to the nearest 0.1 Hz.

#### Synthesis of Catalyst

Diphenyl diselenide (**1**) is commercially available (purchased from Sigma-Aldrich (St. Louis, MO, USA) and was used as received.

Diselenobis benzoic acid (**38**) was synthetized as follows. In a Schlenk tube, elemental selenium (699.2 mg, 8.74 mmol) was added and filled with argon. Separately, in a test tube at 0 °C, a solution of KOH (735.6 mg, 13.11 mmol) and KBH_4_ (59.33 mg, 1.10 mmol) in 2 mL of distilled water was prepared and added at once to the Schlenk tube. The reaction was sonicated for 30 min. In the meantime, in a round-bottom flask (50 mL) with three necks, anthranilic acid (1.199 g, 8.74 mmol) was solubilized in 12 mL of water and 0.65 μL of HCl (37%). In another test tube, NaNO_2_ (724.5 mg, 10.5 mmol) was solubilized in 4 mL of distilled water. Then, the solution was dropped in the round-bottom flask at 0 °C and the reaction was stirred for 30 min. To the 2-carboxybenzenediazonium chloride, the freshly prepared solution of Se_2_K_2_ was added dropwise. The reaction mixture was allowed to reach room temperature and then was stirred for 2 h at 90 °C. Later, it was filtrated with Celite using a Buchner funnel and then acidified with 30 mL of HCl 1M. The beige precipitate was filtered with a Hirsh funnel and washed with hot methanol. Then, it was concentrated under reduced pressure and the solid obtained was crystallized in 1–4 dioxane to give 1.341 mg (3.33 mmol, 76% yield) of a white–pink crystal, m.p.: 290–292 °C (289–292 °C ref. [21]). **^1^H-NMR (DMSO-d_6_**, **600 MHz**, **298 K**, **TMS):** δ 8.04 (d, *J* = 7.7 Hz, 2H), 7.67 (d, *J* = 8.0 Hz, 2H), 7.49 (t, *J =* 7.5 Hz, 2H), 7.40 (t, *J =* 7.4 Hz, 2H) ppm; **^13^C-NMR (DMSO-d_6_**, **150 MHz):** δ 169.0, 134.0, 133.9, 129.6, 129.3, 126.9, 66.8 ppm; **^77^Se-NMR (DMSO-d_6_**, **114 MHz):** δ 439.28 ppm.

### 3.2. Oxidation Reaction of Model Compound

To a solution of selected substrate (2.65 mmol) in H_2_O (5 mL), the catalyst (10 mol% equiv. or 20 mol% equiv.) was added. Then, to the reaction mixture, the oxidant (10 mol. equiv. or 30 mol. equiv.) was added, and the reaction was allowed to be stirred for the time and under the conditions reported in the tables. The reaction was extracted with EtOAc (20 mL × 3), and the organic phase was washed with brine and dried over sodium sulfate, filtrated and concentrated under reduced pressure. In some cases, the products were analyzed after esterification of the acids obtained by oxidation. Esterification was performed as follows: 15 mL of a solution of CH_2_N_2_ 0.1 M was directly added to the crude of the selected reaction solubilized in Et_2_O (20 mL) and the reaction was monitored by TLC (EP:EtOAc 20%). After complete consumption of the starting material, the reaction was washed with a basic solution (NaHCO_3_ 10%) and extracted with EtOAc (20 mL × 3). The organic phase was washed with brine and dried over sodium sulfate, filtrated and concentrated under reduced pressure.

### 3.3. Olive Mill Waste Water Oxidation and Quantification

The reactions were performed in 300 mL flasks and stirred with Teflon-coated magnetic stirring bars. The solvents and reagents were used as received unless otherwise noted. The NMR experiments were performed at 25 °C on a Bruker Advance Neo spectrometer operating at 600 MHz with a CryoProbe Prodigy and sample jet, for ^1^H, 100.62 MHz for ^13^C and 114 MHz for ^77^Se. The ^1^H, ^13^C and ^77^Se chemical shifts (δ) are reported in parts per million (ppm) and they are relative to TMS 0.0 ppm and the residual solvent peak of CDCl_3_ at δ 7.26 and δ 77.00 in the ^1^H and ^13^C NMR, respectively. The quantification experiments were performed using a UV/Vis spectrophotometer (Tecan Infinite 200Pro, in a Greiner 96/384 flat-bottom transparent polystyrene). Centrifugation was performed using the centrifuge NF 800R.

#### 3.3.1. Oxidation of OMW

After filtration under a vacuum using cotton, 100 mL of OMW purchased from EcoTech Engineering s.p.a during the 2021–2022 season was transferred into a 300 mL flask and to this was added the selected amount of H_2_O_2_ corresponding to 10 mL, 1 mL, and 0.1 mL of a 30% diluted solution of H_2_O_2_. To reach the same final volume of 110 mL for each sample, H_2_O was added. Specifically, 9.0 mL was added to the OMW oxidized with 1 mL of oxidant and 9.9 mL was added to the OMW oxidized with 0.1 mL of oxidant. Then, the catalyst in the selected amount (611 mg, 61.1 mg and 6.1 mg) was added and the reaction was allowed to be stirred for 24 h under the desired conditions: room conditions, Blue LED, Green LED or UV irradiation.

#### 3.3.2. F-C Quantification of OMW

The F-C quantification was carried out using the protocol developed by Samara et al. [49] and adapted to our samples. Calibration curve: a solution of gallic acid with a concentration of 8.75 mg/mL was prepared by dissolving 17.50 mg of the standard in 2 mL of deionized water. From this, 142.9 μL was picked up and diluted to 5 mL, obtaining an intermediate stock solution with a concentration of 250 μg/mL. At this point, seven solutions were prepared as follows:

(a) First, 0.1 mL of the stock solution (250 μg/mL) was picked up and diluted to 1 mL, obtaining a solution with a concentration of 25 μg/mL.
(b) Second, 0.08 mL of the stock solution (250 μg/mL) was picked up and diluted to 1 mL, obtaining a solution with a concentration of 20 μg/mL.
(c) Third, 0.05 mL of the stock solution (250 μg/mL) was picked up and diluted to 1 mL, obtaining a solution with a concentration of 12.5 μg/mL.
(d) Fourth, 0.03 mL of the stock solution (250 μg/mL) was picked up and diluted to 1 mL, obtaining a solution with a concentration of 7.5 μg/mL.
(e) Fifth, 0.020 mL of the stock solution (250 μg/mL) was picked up and diluted to 1 mL, obtaining a solution with a concentration of 5 μg/mL.
(f) Sixth, 0.015 mL of the stock solution (250 μg/mL) was picked up and diluted to 1 mL, obtaining a solution with a concentration of 3.75 μg/mL.
(g) Seventh, 0.010 mL of the stock solution (250 μg/mL) was picked up and diluted to 1 mL, obtaining a solution with a concentration of 2.5 μg/mL.

An aliquot of 0.650 mL of each solution was then placed in an Eppendorf tube and 0.300 mL of 20% (*w*/*v*) Na_2_CO_3_ solution was added. A volume of 0.05 mL of 1:1 diluted F-C reagent was then added, and the samples were incubated for 30 min at room temperature, avoiding direct contact with light. Preparation of the samples: the OMW samples were diluted 5000 times with deionized water and were filtrated using 0.45 μm Teflon filters (Sample NT was 10,000-fold diluted). Samples of 0.650 mL of the diluted OMW were then placed in an Eppendorf tube and 0.300 mL of 20% (*w*/*v*) Na_2_CO_3_ solution were added. A volume of 0.05 mL of 1:1 diluted F-C reagent was then added, and the samples were incubated for 30 min at room temperature, avoiding direct contact with light. Both the solutions prepared for the calibration curve and the samples were analyzed in triplicate after 30 min and the absorbance was measured at 725 nm, using the Tecan Infinite 200Pro.

#### 3.3.3. Polyphenol Extraction

The OMW samples were filtered under a vacuum to eliminate the solid components using cotton and then centrifuged at 5000 rpm for 15 min at 15 °C. After decantation, delipidation was performed by adding n-hexane to the liquid part of the sample (1:1, *_v_*_/*v*_) and stirring for one hour. Then, the two phases were separated by centrifugating at 5000 rpm for 15 min at 15 °C. The extraction of phenolic compounds from the aqueous phase was performed using ethyl acetate (1:2, *_v_*_/*v*_), and the sample mixture was stirred in a flask for 30 min. The organic phase, which contains the phenolic compounds, was separated from the aqueous phase through separating funnel. Finally, ethyl acetate was concentrated under a vacuum using a rotary evaporator [44]. The viscous extract obtained was successively quantified using a Tecan Infinite 200Pro.

#### 3.3.4. Acidic Polyphenol Extraction

After under-vacuum filtration was carried out using cotton, 10 mL of OMW sample was acidified to pH 2 with a solution of HCl 10%. Then, delipidation was performed by adding 15 mL of n-hexane and the two-phase system was shaken through a separating funnel and centrifuged for 5 min at 3000 rpm. The two phases were separated, and this procedure was repeated successively two times. Then, the extraction of phenolic compounds was performed using 10 mL of ethyl acetate and the two-phase system was shaken through a separating funnel and centrifuged for 5 min at 3000 rpm. This procedure was repeated four times, and the organic phase was concentrated under reduced pressure [45]. The viscous extract obtained was successively quantified using a Tecan Infinite 200Pro.

#### 3.3.5. Extracted Polyphenol Quantification

Polyphenol quantification has been performed according to the literature [50]. Calibration curve: 200 mg of gallic acid was dissolved in a volumetric flask and diluted to 100 mL with deionized water. Aliquots of 2.630, 4, 7, 10, 13 and 16 mL were picked up and transferred in a 100 mL volumetric flask and diluted to 100 mL. From these solutions, aliquots of 20 mL were taken and successively diluted to 100 mL. From these, 8 mL of solution was transferred into a 100 mL volumetric flask and 4 mL of 2N Folin–Ciocalteu reagent and 40 mL of deionized water were added and then volumetrically diluted to 100 mL with 10.75% (*w*/*v*) of an anhydrous Na_2_CO_3_ solution, reaching the concentration of 0.84, 1.28, 2.24, 3.20, 4.16 and 5.12 μg/mL of gallic acid. Sample preparation: the extract (26.0 mg) was transferred to a 25 mL volumetric flask and diluted with deionized water. Then, 5 mL of this solution was transferred into another volumetric flask and the volume of 25 mL was reached using deionized water (Sample NT was prepared in a twofold diluted solution; Samples S1, S3, and S4 were prepared in a twofold concentrated solution). Then, a solution was prepared by adding 2 mL of the sample, 1 mL of 2N Folin–Ciocalteu reagent, and 10 mL of water and volumetrically diluted to 25 mL with 10.75% (*w*/*v*) of an anhydrous Na_2_CO_3_ solution. Both the solutions prepared for the calibration curve and the samples were analyzed in triplicate after 30 min and the absorbance was measured at 765 nm, using the Tecan Infinite 200Pro.

#### 3.3.6. Polyphenol Quantification by ^1^H-NMR

The ^1^H-NMR quantification was performed by adding 0.040 mL of a solution of N,N-dimethylformamide analytical standard prepared by adding 0.051 mL of DMF and volumetrically diluting it to 5 mL with D_2_O, in a NMR tube where previously were transferred 0.450 mL of the OMW sample and 0.050 mL of D_2_O. All the spectra were recorded in water suppression mode at room temperature with 8 scans.

#### 3.3.7. Catalyst Removal by Filtration

Here, 200 mL of treated and untreated OMW were fluxed in two columns packed with 6 alternate layers of AmberLite^®^ IRA-900 and SiO_2_ (60–120 mesh), recovering 170 mL of OMW. The aliquot was analyzed by ^1^H-NMR (Supplementary Materials Figures S15 and S16).

### 3.4. Acute Toxicity—Microtox^®^ 81.9% Basic Test

This study aimed to assess the acute toxicity of OMW using *Aliivibrio fischeri* bacteria. Before testing, the freeze-dried bacterial culture was rehydrated with a reconstitution solution. Given that *A. fischeri* is a marine organism, the osmotic pressure of the samples was adjusted by adding a concentrated salt solution (22% NaCl in deionized water) to achieve a final salinity of 2%. The acute toxicity was evaluated at 5 and 15 min, expressed as the EC_50_ values, which were determined using the 81.9% basic test protocol in the MicrotoxOmni software, version 4.2. The 95% confidence intervals were automatically calculated by the software through regression analysis.

The EC_50_ value, or the half-maximal effective concentration, is the concentration of a substance that induces a 50% effect in a biological system. It is commonly used to measure the potency or toxicity of a substance, with lower EC_50_ values indicating higher toxicity or effectiveness. The EC_50_ value helps to compare the relative potency of different substances in causing a specific biological response.

## 4. Conclusions

In this manuscript, we have shown that seleno-organic catalysts and hydrogen peroxide can be used in the oxidation of OMW, producing a degradation and, consequently, a decrease in the content of total polyphenols, which are considered responsible for the phytotoxicity of water recovered from olive oil processing and production processes. This study of the effects of oxidative processes on model compounds allowed the optimization of a protocol for the effective removal of the total polyphenols. Toxicological analyses revealed the need to completely remove the oxidizing system (catalyst and peroxide), leading us to show that when this is possible, the toxicity can be reduced by more than 200-fold. The system reported in this work, however, still shows difficult scalability, so studies are currently underway to develop new heterogeneous catalysts that can be more easily removed and hopefully reused.

## Figures and Tables

**Table 1:** Oxidation of **21**.

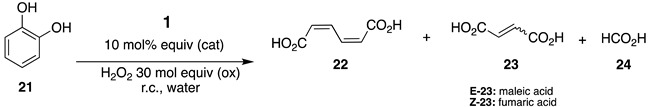								
Entry	Spectrum	Time	NMR Conversion (%)	NMR Yield (%)	22 (%)	E-23 (%)	Z-23 (%)	24 (%)
1	S1-A ^[a]^	30 min	100	99	84	14	2	0
2	S1-B	1 h	100	65	66	27	4	3
3	S1-C	3 h	100	36	74	18	7	1
4	S1-D	6 h	100	35	63	24	11	2
5	S1-E ^[b]^	24 h	100	17	46	30	23	1

^[a]^ Unless otherwise indicated, the reactions were performed with 0.265 mmol of **21** in 5 mL of H_2_O, 30 molar equivalents of H_2_O_2_ and 10 mol% equivalents of **1**; ^[b]^ liberation of CO_2_ was detected using a handmade trap filled with barite water.

**Table 2:** OMW model compounds’ oxidation.

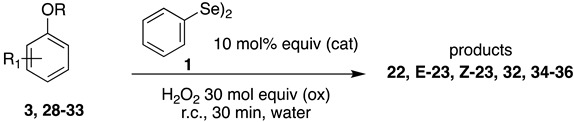				
Entry ^[a]^	Substrate	Conversion (%)	Products (Ratio)	NMR Yield (%)
1	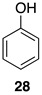	100	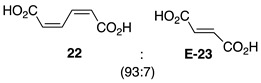	73
2	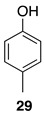	80	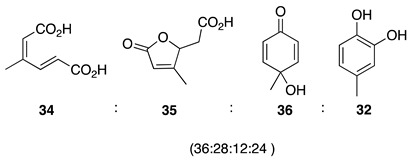	55
3	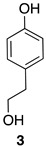	0	-	-
4	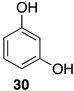	100	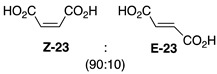	n.c. ^[b]^
5	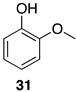	100	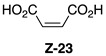	n.c. ^[b]^
6	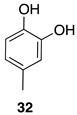	100	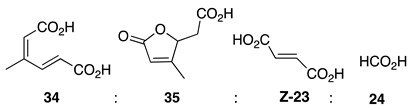	89
7	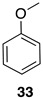	100	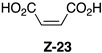	n.c. ^[b]^

^[a]^ Unless otherwise indicated, the reactions were performed with 0.265 mmol of **substrate** in 5 mL of H_2_O, 30 molar equivalents of H_2_O_2_ and 10 mol% equivalents of **1** ^[b]^ n.c.: not calculable because of the presence of non-identified species.

**Table 3:** Phenols concentration before and after oxidation when applying the maximum amounts of the catalyst and oxidant.

Entry	3		2		37		TP		Decrease ^[b]^ (%)
	(μmol) ^[a]^	(mg/mL)	(μmol) ^[a]^	(mg/mL)	(μmol) ^[a]^	(mg/mL)	(μmol) ^[a]^	(mg/mL)	
**SNT**	1.47	0.45	8.48	2.91	1.80	1.35	11.75	4.70	-
**SMAX**	0.91	0.28	4.46	1.53	0.63	0.47	6.0	2.28	49
**S1**	0.81	0.25	0	0	0.11	0.08	0.92	0.33	92
**S2**	0.47	0.14	0	0	0	0	0.47	0.14	96
**S3**	0.42	0.13	0	0	0.21	0.16	0.63	0.29	95
**S4**	0.71	0.22	0	0	0.11	0.08	0.82	0.30	93

^[a]^ μmol in 450 μL; ^[b]^ calculated on TP μmol.

**Table 4:** Phenols concentration before and after oxidation when applying the intermediate amounts of the catalyst and oxidant.

Entry	3		2		37		TP		Decrease ^[b]^ (%)
	(μmol) ^[a]^	(mg/mL)	(μmol) ^[a]^	(mg/mL)	(μmol) ^[a]^	(mg/mL)	(μmol) ^[a]^	(mg/mL)	
**SNT**	1.47	0.45	8.48	2.91	1.80	1.35	11.75	4.70	-
**SMED**	1.10	0.34	5.30	1.82	1.75	1.31	8.15	3.6	31
**S5**	1.02	0.31	4.09	1.40	1.57	1.17	6.68	2.89	43
**S6**	0.94	0.29	3.94	1.35	1.3	0.97	6.18	2.61	47
**S7**	1.02	0.31	5.30	1.82	1.59	1.19	7.91	3.32	33
**S8**	1.03	0.32	5.04	1.73	1.41	1.05	7.48	3.10	36

^[a]^ μmol in 450 μL; ^[b]^ calculated on TP μmol.

**Table 5:** Phenols concentration before and after oxidation when applying the minimum amounts of the catalyst and oxidant.

Entry	3		2		37		TP		Reduction ^[b]^ (%)
	(μmol) ^[a]^	(mg/mL)	(μmol) ^[a]^	(mg/mL)	(μmol) ^[a]^	(mg/mL)	(μmol) ^[a]^	(mg/mL)	
**SNT**	1.47	0.45	8.48	2.91	1.80	1.35	11.75	4.70	-
**SMIN**	1.27	0.39	5.62	1.93	1.48	1.11	8.37	3.42	29
**S9**	1.07	0.33	4.88	1.67	1.36	1.02	7.31	3.02	38
**S10**	1.10	0.34	5.49	1.88	1.37	1.02	7.96	3.24	32
**S11**	1.06	0.33	5.61	1.92	1.54	1.15	8.21	3.40	30
**S12**	1.16	0.36	5.10	1.75	1.53	1.14	7.79	3.25	34

^[a]^ μmol in 450 μL; ^[b]^ calculated on TP μmol.

**Table 6:** Microtox^®^ EC_50_ values (mg/L) of the exposure of the luminescent marine bacteria *A. fischeri* to the tested substances with the respective 95% confidence limits (in brackets) obtained in the fit of the data after 5 min.

Sample	EC_50_ [mg/L]	Determination Coefficient (R^2^)
**SNT**	0.0886 (0.0386–0.2029)	0.09191
**S1**	0.1163 (0.0902–0.1501)	0.9938
**S2**	0.0965 (0.0671–0.1390)	0.9623
**S3**	0.1569 (0.1085–0.2269)	0.9668
**S4**	0.1150 (0.1062–0.1245)	0.9981
**S5**	0.1261 (0.1089–0.1461)	0.9623
**S6**	0.2019 (0.1123–0.3631)	0.9603
**S7**	0.2190 (0.1376–0.3486)	0.9352
**S8**	0.2054 (0.1329–0.3174)	0.9446
**S9**	0.2087 (0.1431–0.3044)	0.9580
**S10**	0.3321 (0.2694–0.4109)	0.9833
**S11**	0.3612 (0.2724–0.4791)	0.9705
**S12**	0.2761 (0.2362–0.3228)	0.9914

**Table 7:** Microtox^®^ EC_50_ values (mg/L) of the exposure of the luminescent marine bacteria *A. fischeri* to the tested substances with the respective 95% confidence limits (in brackets) obtained in the fit of the data after 5 min. SNT-DSBA: OMW non-treated; S1-DSBA: OMW treated with H_2_O_2_ (10% *_v_*_/*v*_) + DSBA (0.6% *_w/w_*) under room conditions; S2-DSBA: OMW after catalyst removal; S3-DSBA: OMW after catalyst removal and UV irradiation.

Sample	EC_50_ [mg/L] (5 Min)	Determination Coefficient (R^2^)
**SNT**	0.0886 (0.0386–0.2029)	0.09191
**S1-DSBA**	1.093 (0.5756–2.076)	0.9488
**S2-DSBA**	6.508 (5.181–8.175)	0.9815
**S3-DSBA**	18.74 (7.107–49.41)	0.9896

## Data Availability

Data are reported in the main manuscript and in the correlated Supplementary Materials and can be additionally requested from the corresponding author.

## Supplementary Materials

The following supporting information can be downloaded at https://www.mdpi.com/article/10.3390/ijms26115192/s1. References [51,52,53,54,55,56,57,58] are cited in the Supplementary Materials.

## Abbreviations

The following abbreviations are used in this manuscript:

OMW	Olive mill wastewater
GPx	Glutathione peroxidase
F-C	Folin–Ciocalteu
TPs	Total phenols
AOPs	Advanced oxidative processes
WAO	Wet air oxidation
NMR	Nuclear magnetic resonance
