# Supplementary material for: Green Chemistry Meets Olive Mill Wastewater: Bioinspired Oxidation of Phenols and Polyphenols Using Selenium Catalysts
Source: Int J Mol Sci. 2025 May 28;26(11):5192. doi: 10.3390/ijms26115192 (PMC12155173; doi:10.3390/ijms26115192)
Supplement: Supplementary file 1 [file ijms-26-05192-s001.zip › ijms-3617742-supplementary.pdf]

Supplementary Materials

Green Chemistry Meets Olive Mill Wastewater: Bioinspired Oxidation of Phenols and Polyphenols Using Selenium Catalysts

Cecilia Scimmi [1], Izabela Szymanek [2], Diana Rogacz [2], Sebastiano Passeri [1], Giulia Patanella [1], Cezary Kozłowski [2], Małgorzata Deska [2], Piotr Richter [2], Jozef Drabowicz [2,3] and Claudio Santi [1,*]

[1] Group of Catalysis, Sinthesis and Organic Green Chemistry, Department of Pharmacuetical Sciences, University of Perugia, via del Liceo 1, 06123 Perugia, Italy; cecilia.scimmi@dottorandi.unipg.it (C.S.); sebastianopasseri1@gmail.com (S.P.); giulia.patanella@studenti.unipg.it (G.P.)

[2] Faculty of Science and Technology, Jan Długosz University in Częstochowa, 13/15 Armii Krajowej Av., 42-200 Częstochowa, Poland; izabela.szymanek@doktorant.ujd.edu.pl (I.S.); d.rogacz@ujd.edu.pl (D.R.); c.kozlowski@ujd.edu.pl (C.K.); m.deska@ujd.edu.pl (M.D.); p.rychter@ujd.edu.pl (P.R.); j.drabowicz@ujd.edu.pl (J.D.)

[3] Division of OrganicChemistry, Center of Molecular and Macromolecular Studies, Polish Academy of Sciences, 112 Sienkiewicza, 90-363 Lodz, Poland

* Correspondence: claudio.santi@unipg.it; Tel.:+39-07-5585-5106

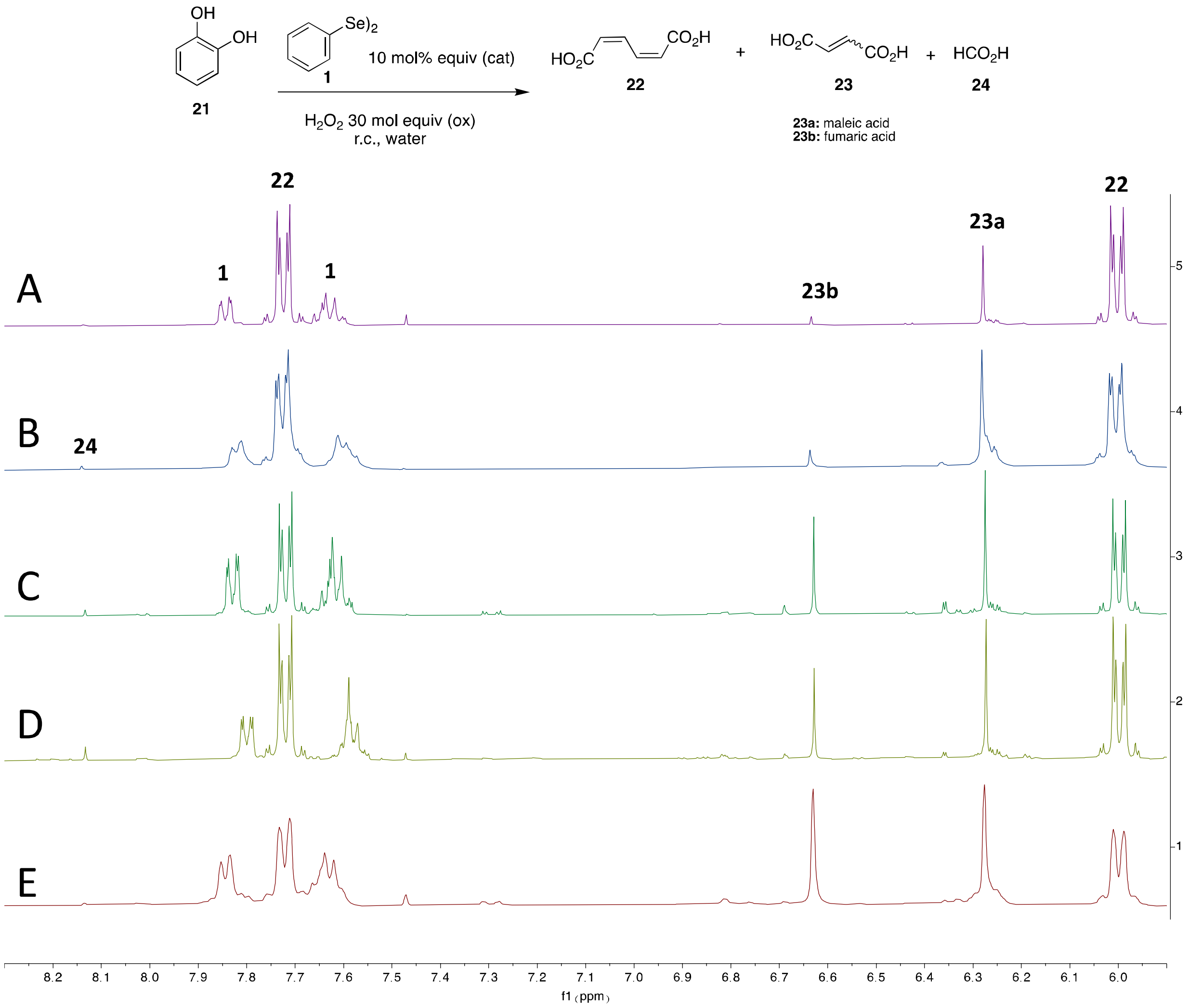

Figure S1. ^{1}H-NMR spectra (in DMSO-d_6) superimposing of room conditions oxidation reactions performed for different reaction times. A: 30 min, B: 1 h, C: 3 h, D: 6 h, E: 24 h [1–4].

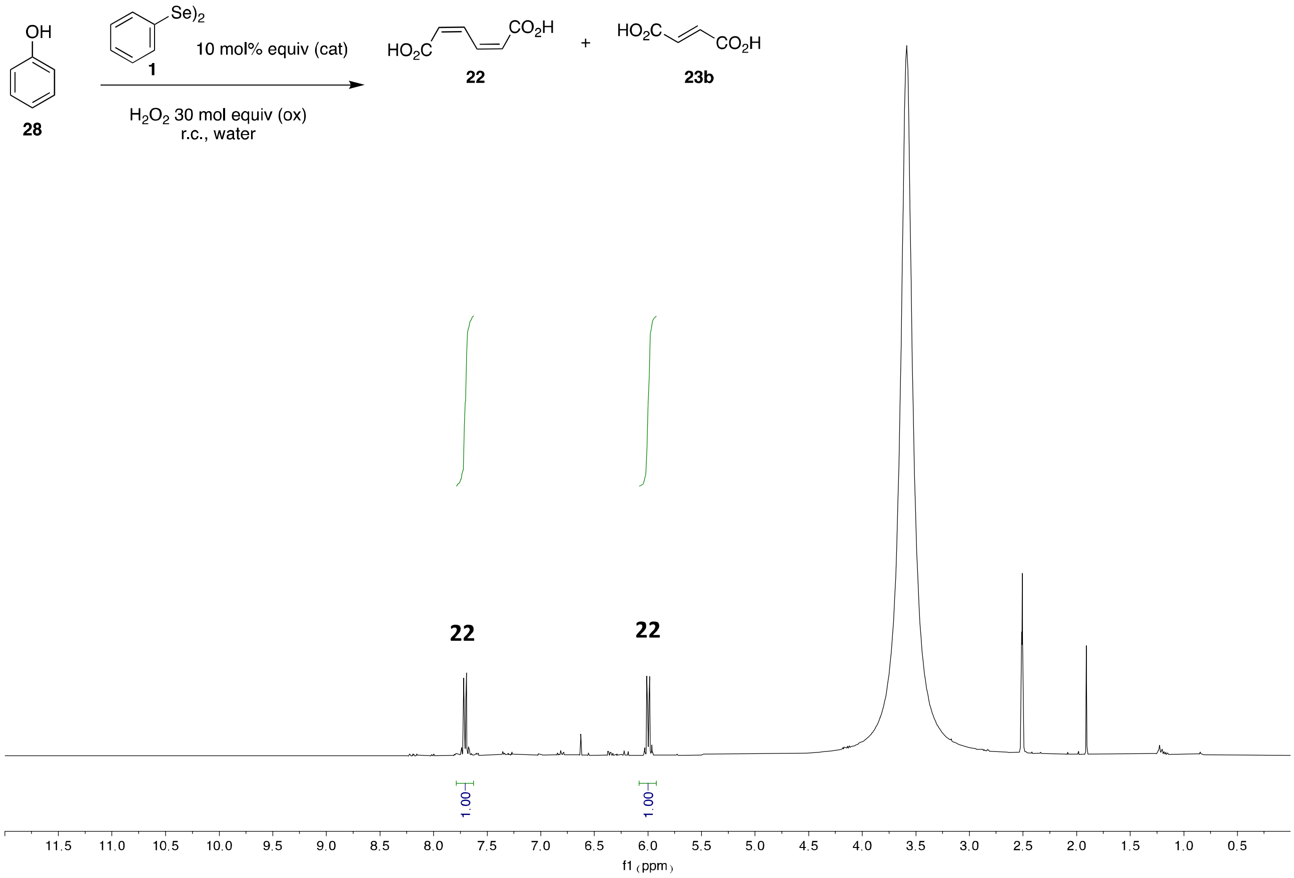

Figure S2. ^{1}H-NMR spectra (in DMSO-d_6) of **28** oxidation [1].

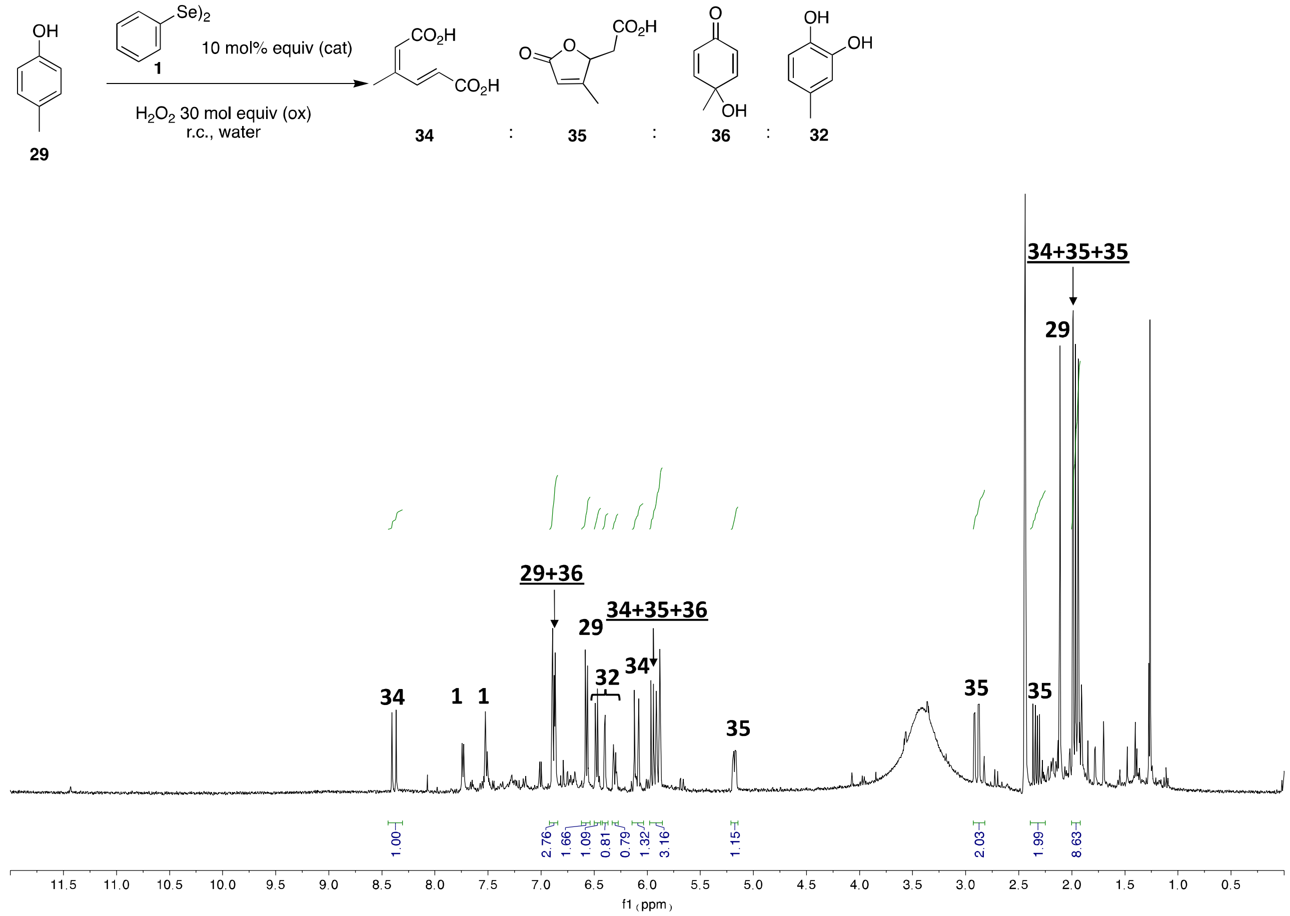

Figure S3. ^{1}H-NMR spectra (in DMSO-d_6) of **29** oxidation [5,6].

Considering that the NMR spectrum of **34** and **35** in DMSO-*d6* are not reported in literature, the formation of **34** and **35** was confirmed after esterification and purification by column chromatography. Physical and spectroscopic date are the following:

Dimethyl (2Z,4E)-3-methylhexa-2,4-dienedioate (34) [7].

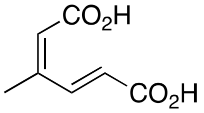

34

Compound **34** was obtained as a yellow oil through the general procedure A and B. It was purified by column chromatography eluting with EP:EtOAc (70:30) yield in 10% yield. **^{1}H-NMR ($CDCl_3$, 400 MHz, 298 K, TMS):** δ 8.55 (dd, *J = 0.9 and 16.1 Hz*, 1H), 6.10 (dd, *J = 0.7 and 16.1 Hz*, 1H), 5.89-5.86 (m, 1H), 3.73 (s, 3H), 3.68 (s, 3H), 1.97 (d, *J = 1.3 Hz*, 3H) ppm; **^{13}C-NMR ($CDCl_3$, 100 MHz):** δ 165.9, 164.7, 146.5, 139.3, 123.1, 122.1, 50.9, 50.5, 19.5 ppm.

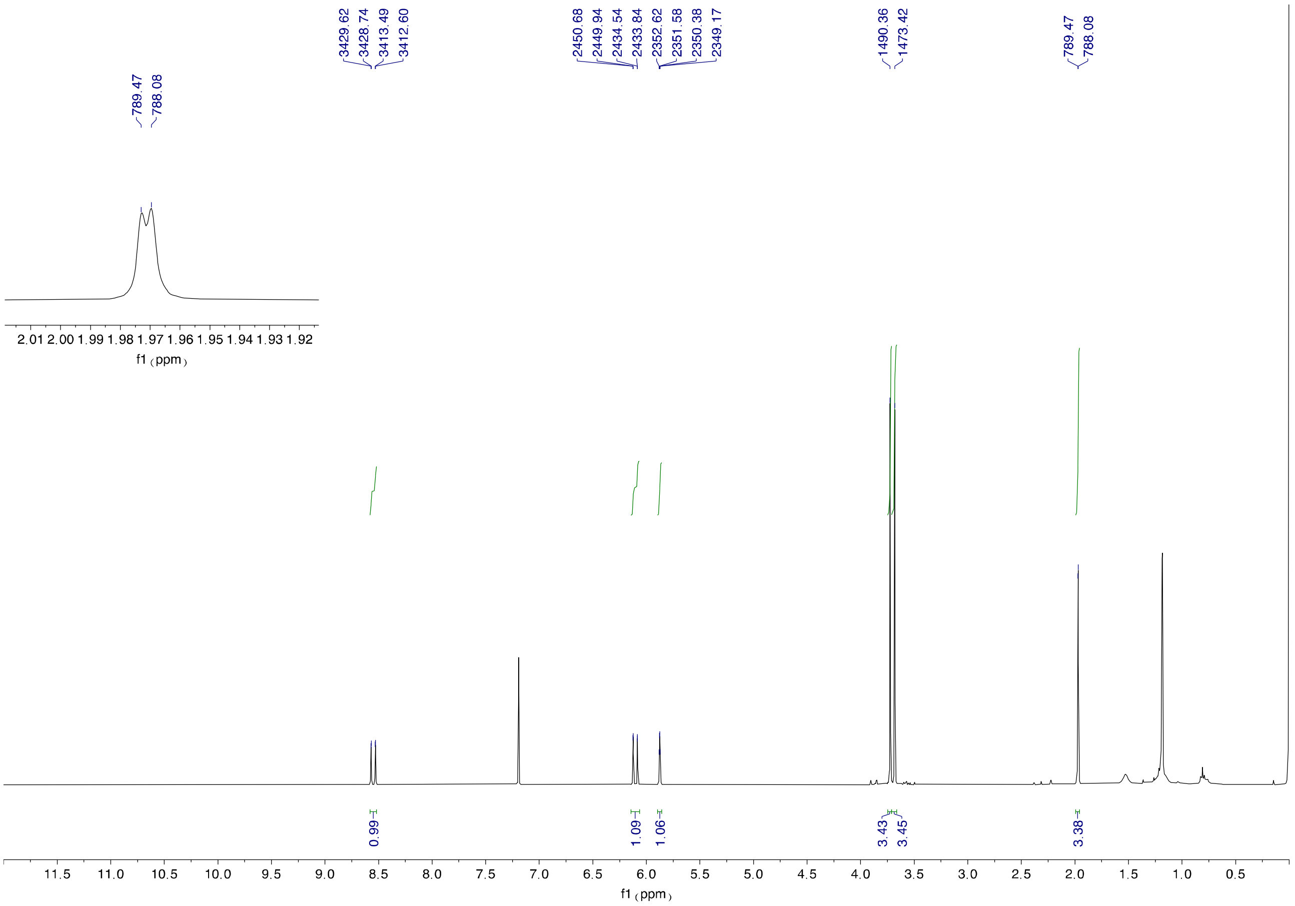

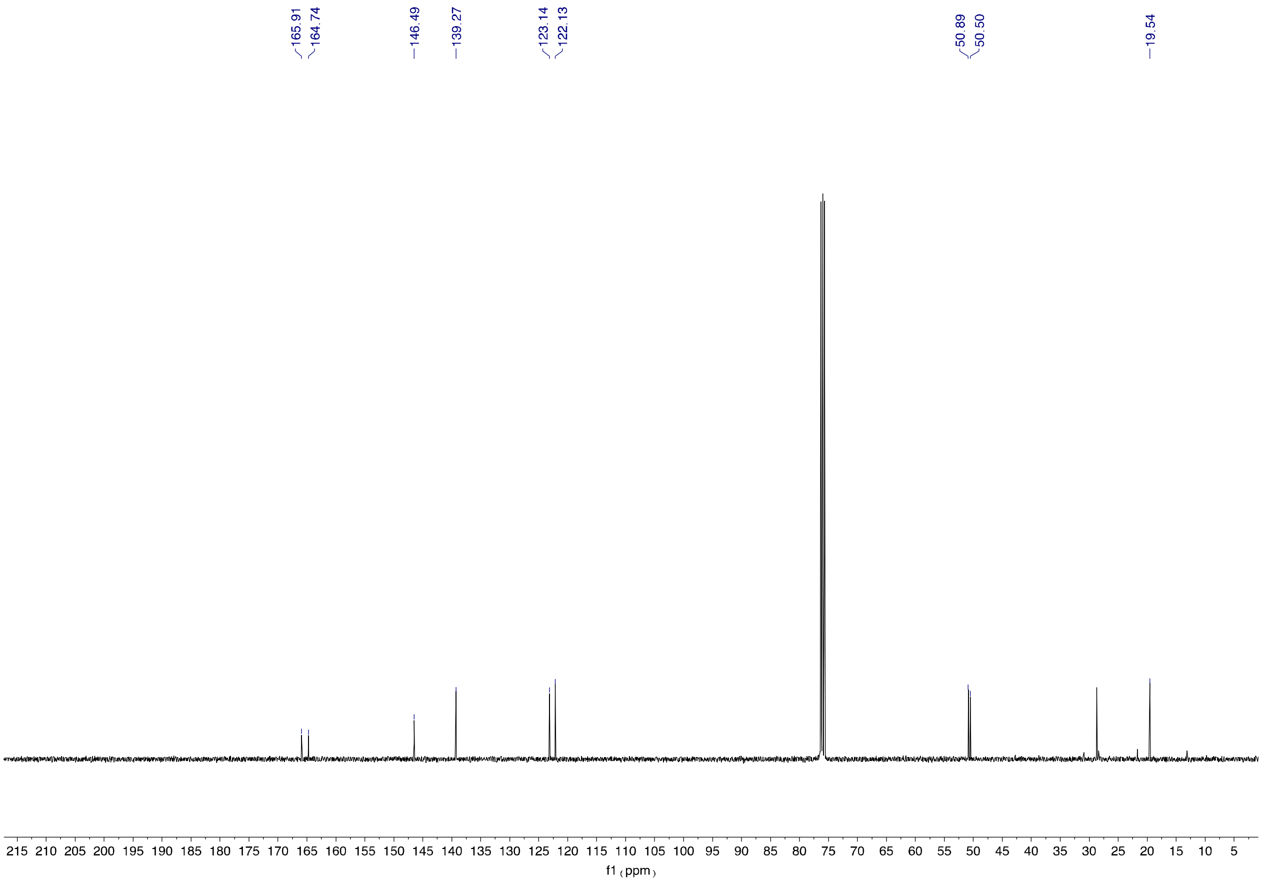

Methyl 2-(3-methyl-5-oxo-2,5-dihydrofuran-2-yl)acetate (35) [7].

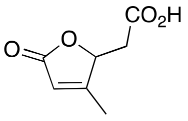

35

Compound **35** was obtained as a yellow liquid through the general procedure A and B. It was purified by column chromatography eluting with EP:EtOAc (70:30) in 9%. **^{1}H-NMR (CDCl₃, 400 MHz, 298 K, TMS):** δ 5.83-5.76 (m, 1H), 5.22-5.14 (m, 1H), 3.68 (s, 3H), 2.73 (dd, *J = 4.7 and 16.0 Hz*, 1H), 2.54 (dd, *J = 7.9 and 16.1 Hz*, 1H), 2.06-1.99 (dd, *J = 0.7 and 1.5 Hz*, 3H) ppm; **^{13}C-NMR (CDCl₃, 100 MHz):** δ 172.2, 169.5, 167.2, 117.7, 80.5, 52.3, 37.1, 13.9 ppm.

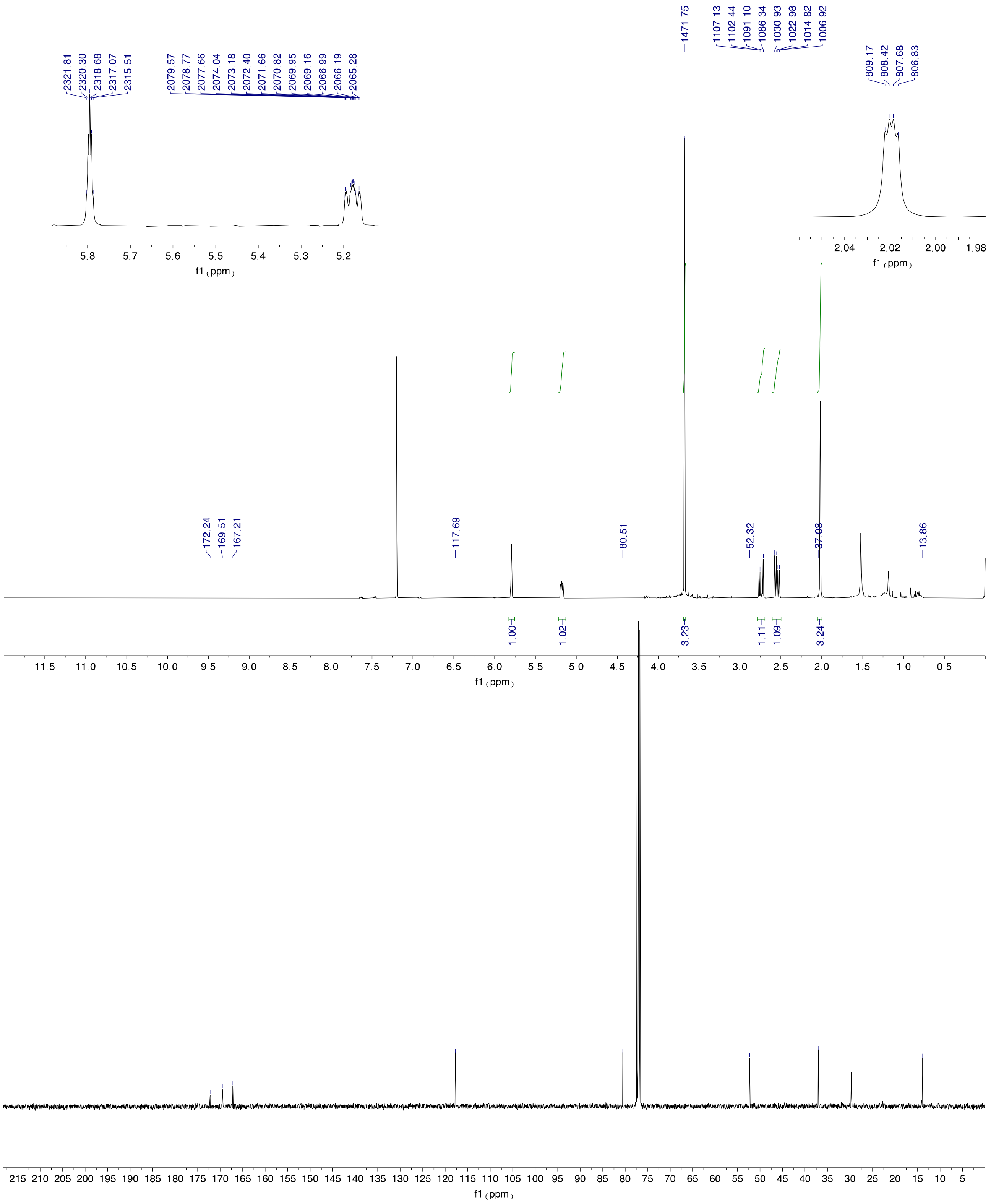

2,2′-diselenobis(benzoic acid) (DSBA)(38) [8].

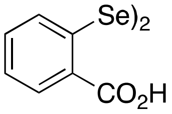
Se)2
CO2H

38

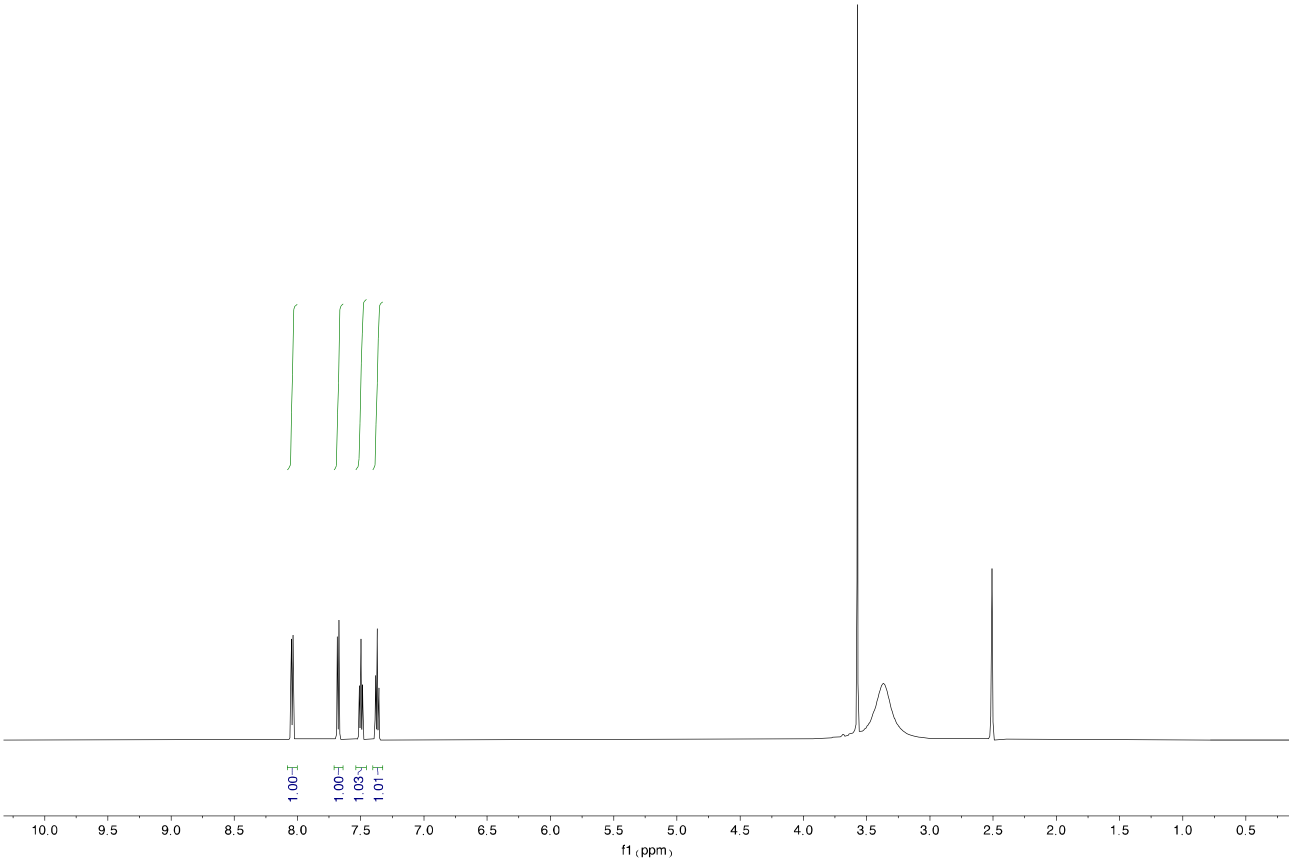
1.00
1.00
1.03
1.01
10.0
9.5
9.0
8.5
8.0
7.5
7.0
6.5
6.0
5.5
5.0
4.5
4.0
3.5
3.0
2.5
2.0
1.5
1.0
0.5
f1 (ppm)

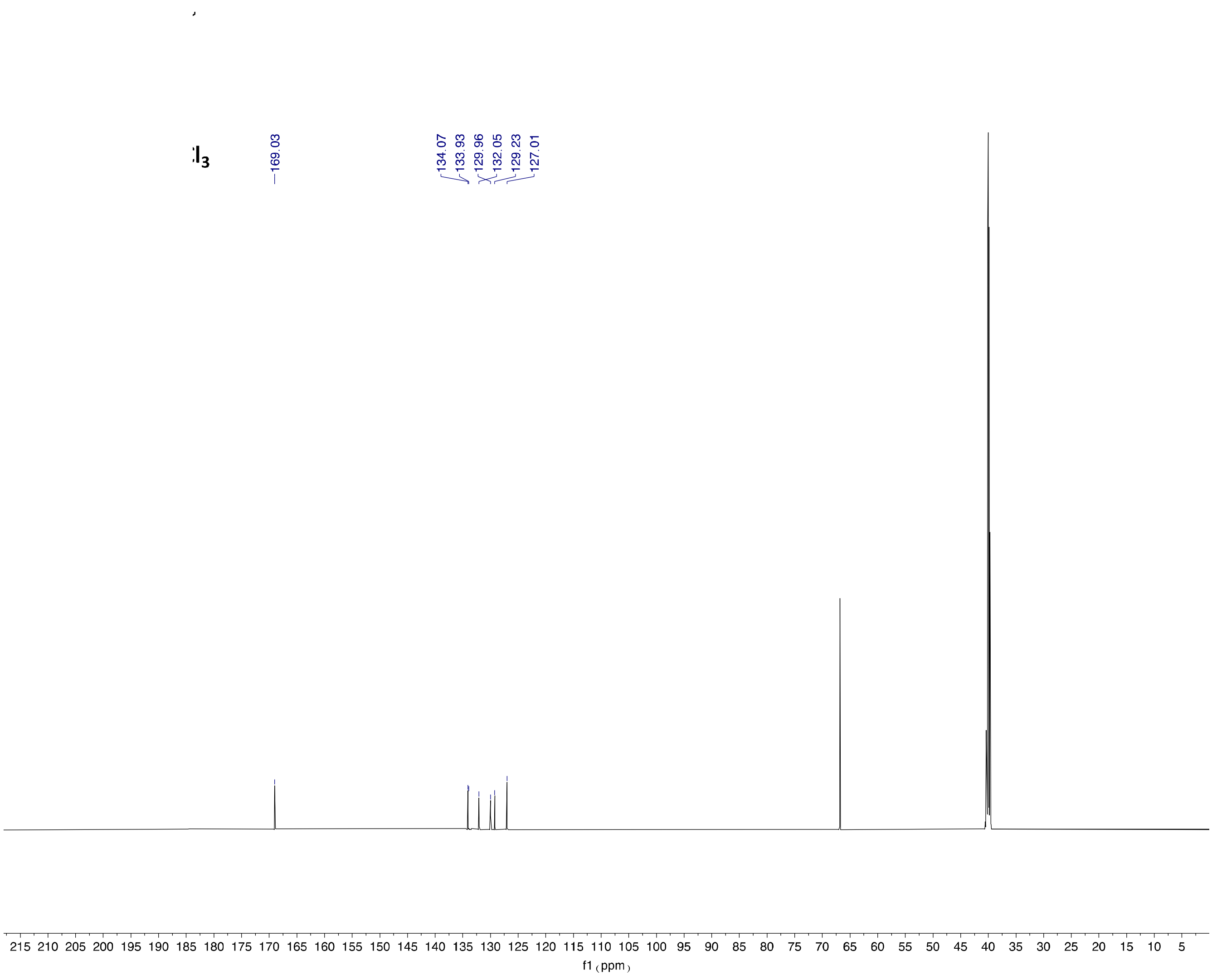

:l₃
169.03
134.07
133.93
129.96
132.05
129.23
127.01
215 210 205 200 195 190 185 180 175 170 165 160 155 150 145 140 135 130 125 120 115 110 105 100 95 90 85 80 75 70 65 60 55 50 45 40 35 30 25 20 15 10 5
f1 (ppm)

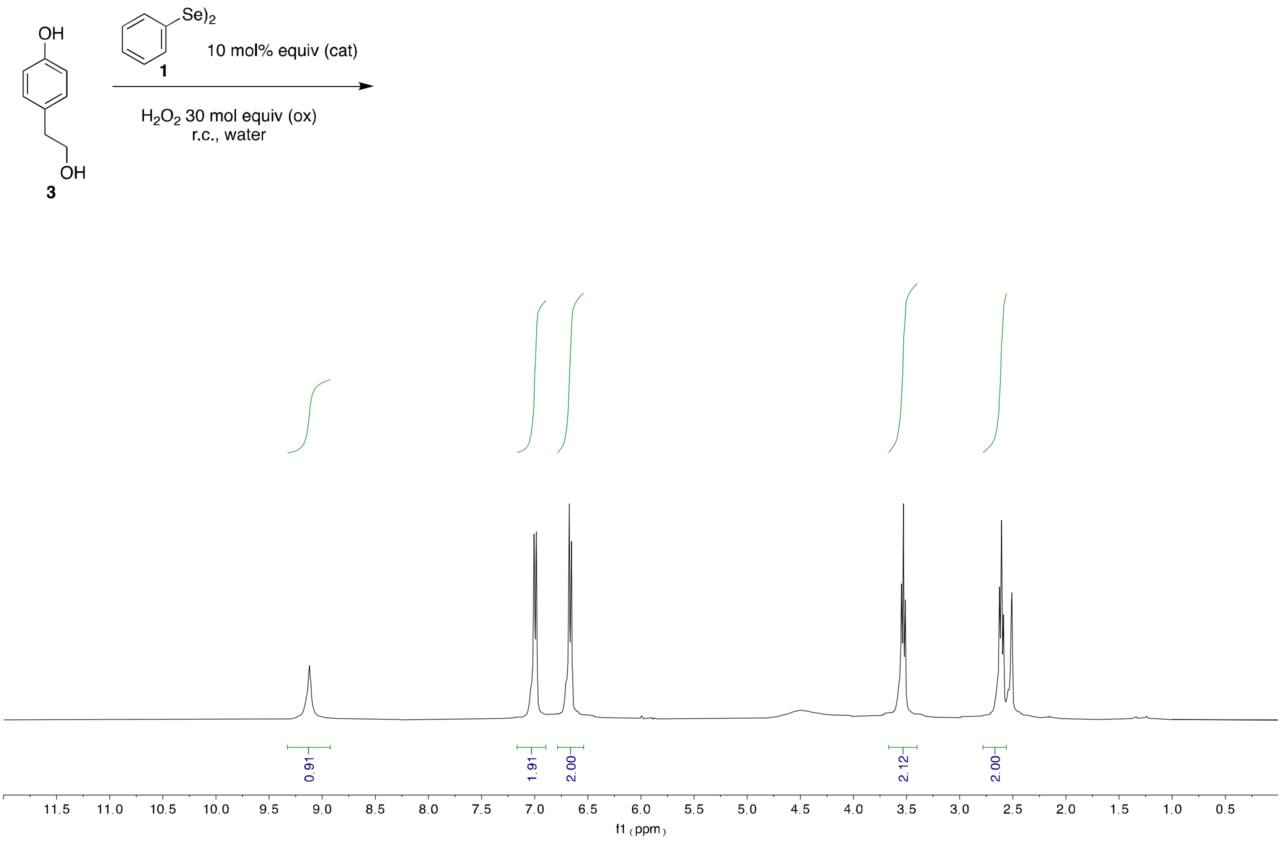

Figure S4. ^{1}H-NMR spectra (in DMSO-d_6) of **3** oxidation [9].

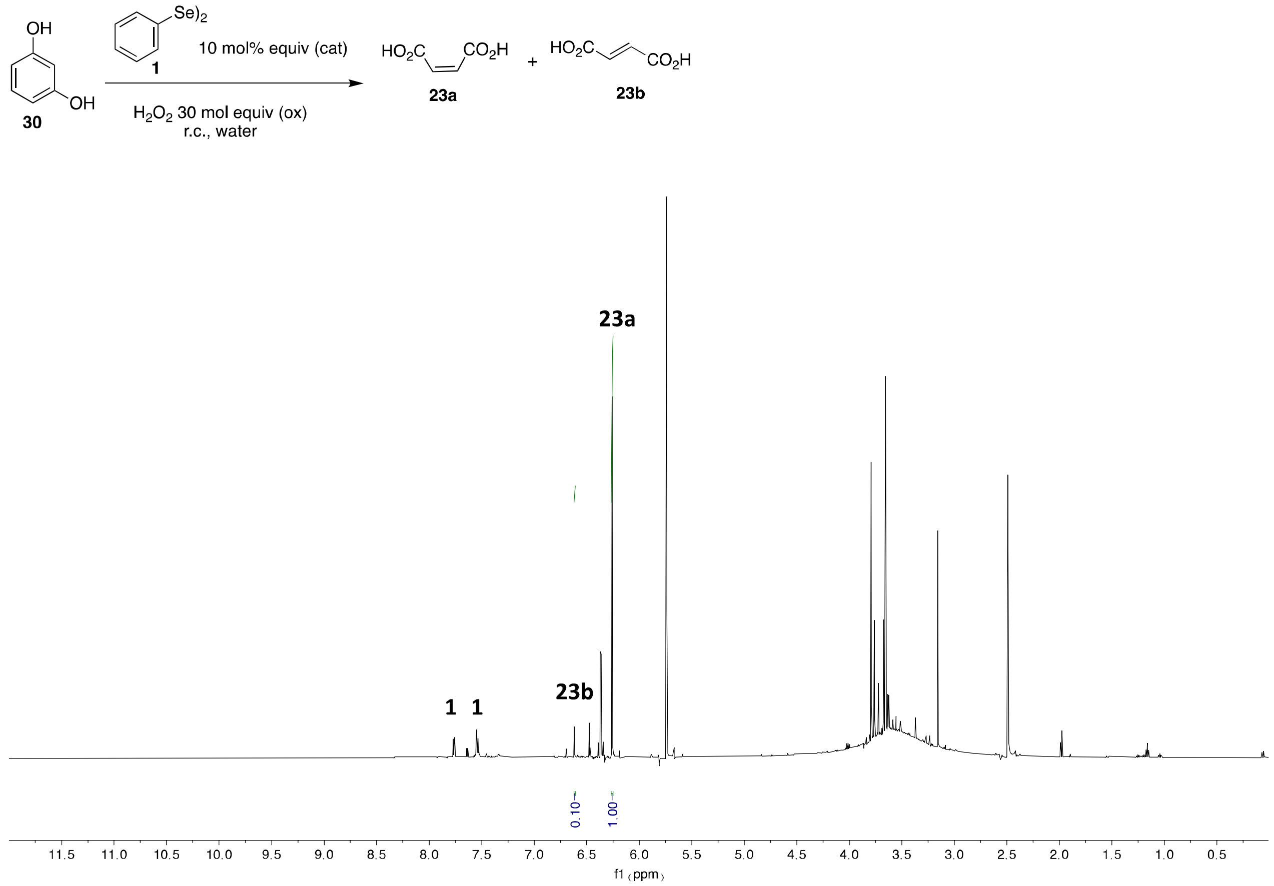

Figure S5. ^{1}H-NMR spectra (in DMSO-d_6) of **30** oxidation.

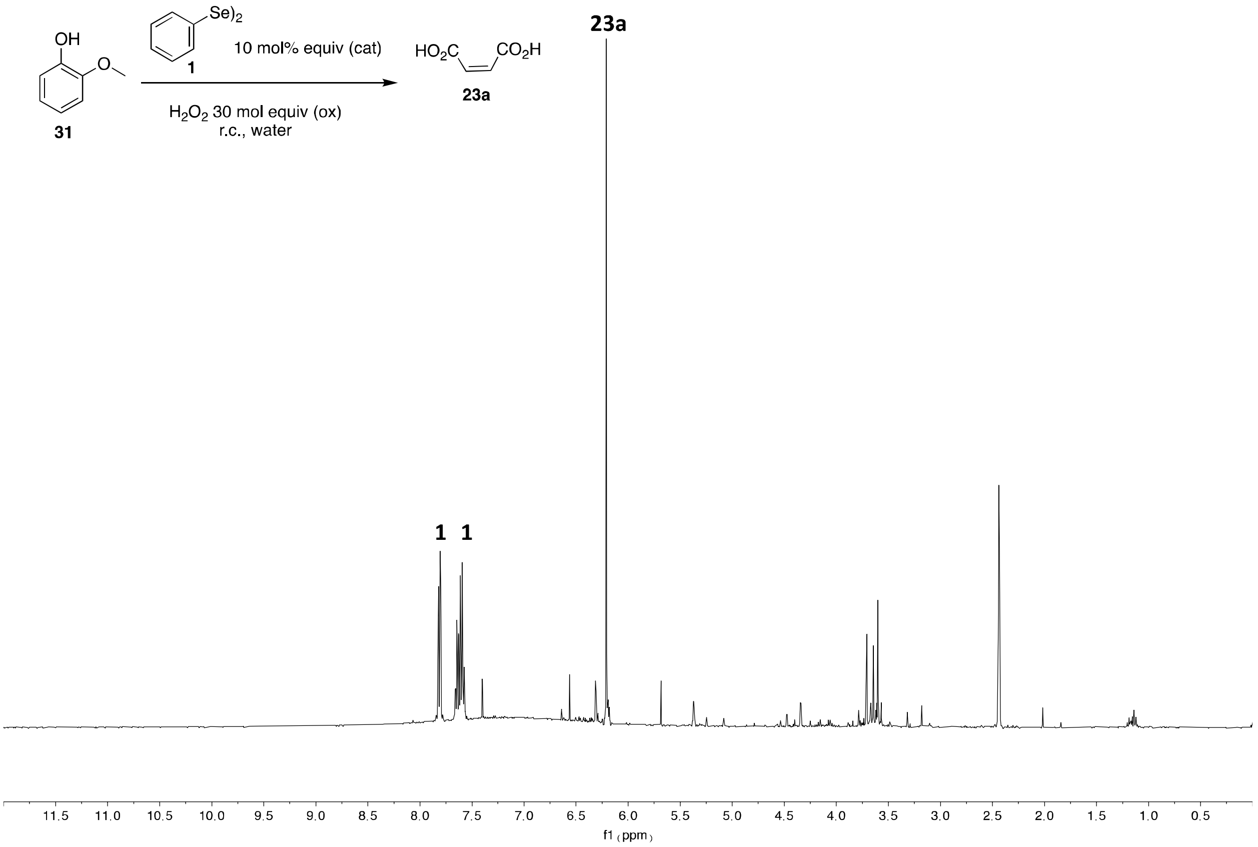

Figure S6. ^{1}H-NMR spectra (in DMSO-d_6) of **31** oxidation.

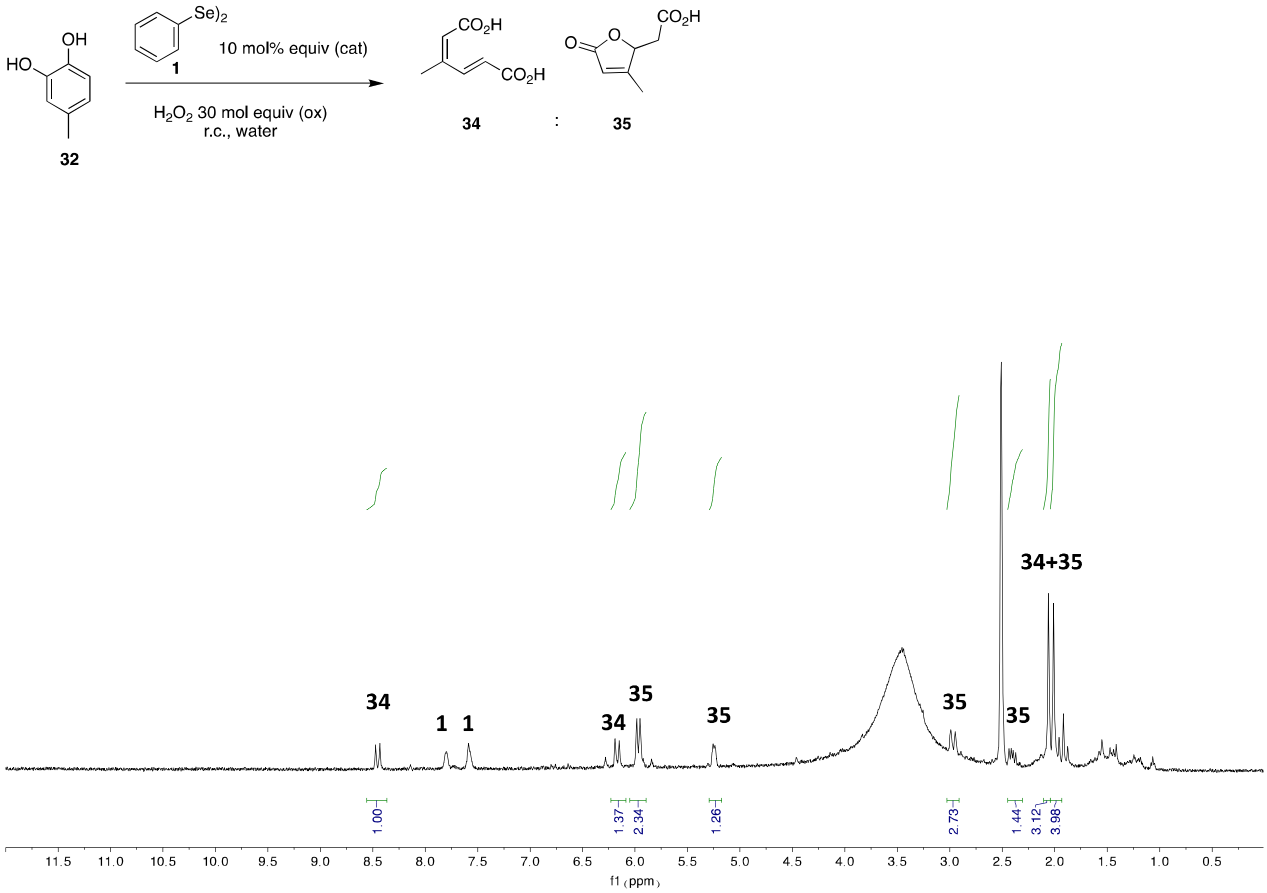

Figure S7. ^{1}H-NMR spectra (in DMSO-d_6) of **32** oxidation.

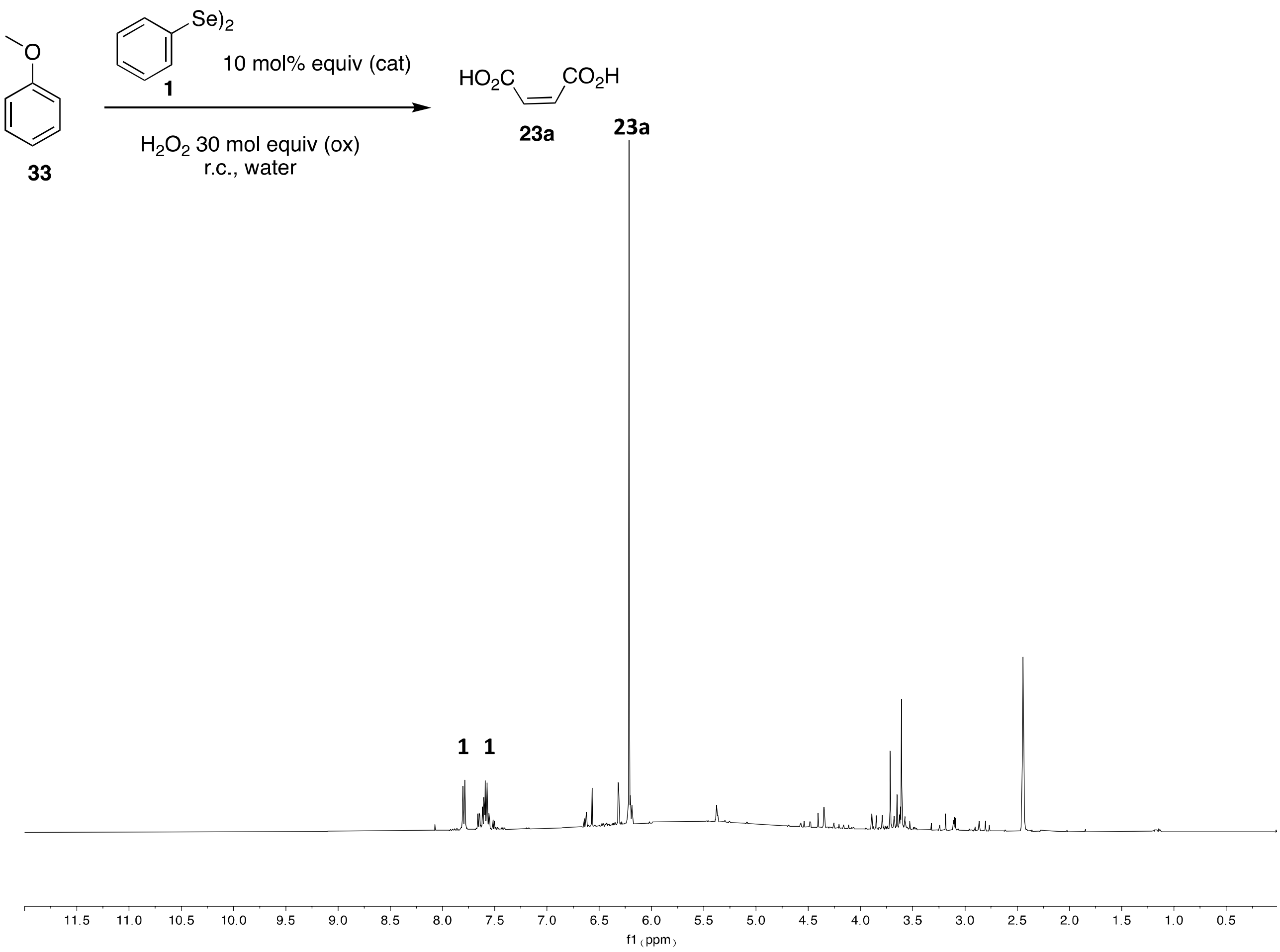

Figure S8. ^{1}H-NMR spectra (in DMSO-d_6) of **33** oxidation.

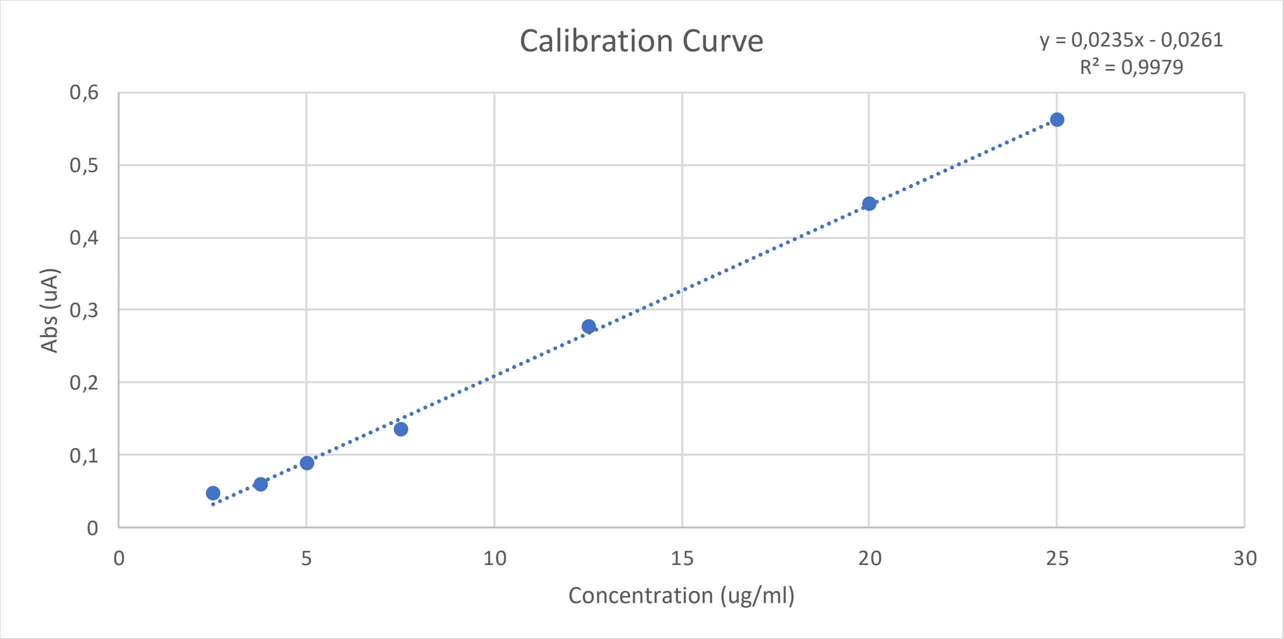

a

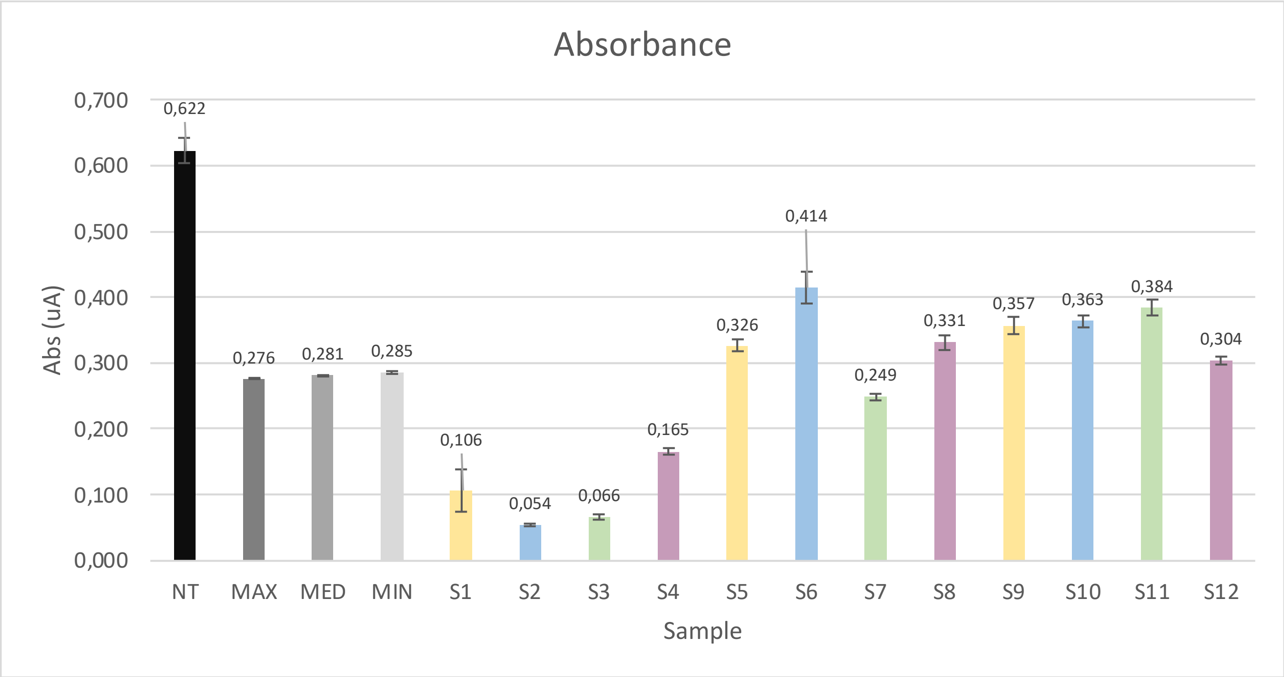

b

Figure S9. (**a**) Calibration curve of F-C quantification carried out with the procedure reported in Materials and Methods in paragraph 3.3.2; (**b**) Absorbance of the samples quantified using the Materials and Methods in paragraph 3.3.2.; Experiments were performed in triplicates and represented as means. Standard deviations are reported as error bar. All the experiments are statistically significant ($p < 0.05$ vs. NT, p values were calculated applying the unpaired Student's t test).

Table S1. Absorbance Means, Standard Deviations and p-value of samples.

**Sample**	Absorbance Means	Standard Deviations	p-Value [a]
NT	0.622	0.019	-
MAX	0.276	0.0012	0.0001
MED	0.281	0.0011	0.0001
MIN	0.285	0.0018	0.0001
S1	0.106	0.033	0.0001
S2	0.054	0.0015	0.0001
S3	0.066	0.0047	0.0001
S4	0.165	0.0048	0.0001
S5	0.326	0.0093	0.0001
S6	0.414	0.0239	0.0003
S7	0.249	0.0052	0.0001
S8	0.331	0.0106	0.0001
S9	0.357	0.0131	0.0001

S10	0.363	0.0093	0.0001
S11	0.384	0.0118	0.0001
S12	0.304	0.0068	0.0001

[a] $p < 0.05$ vs. NT, p values were calculated applying the unpaired Student's *t* test.

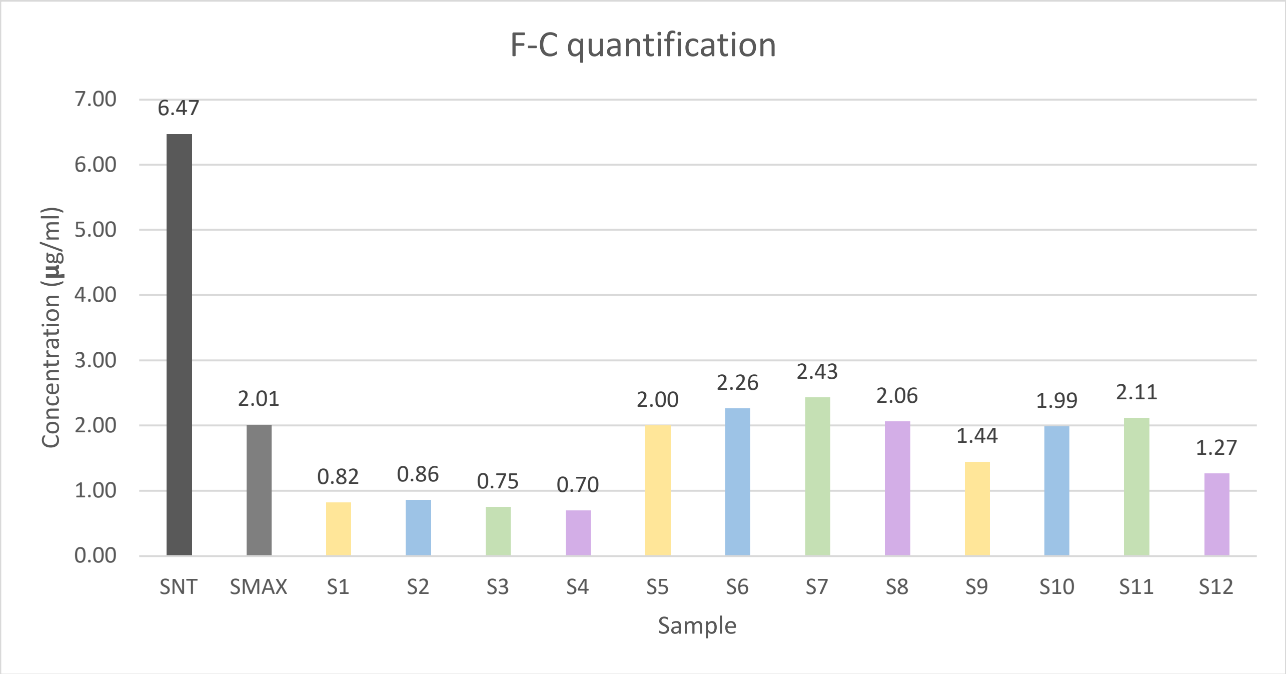

a

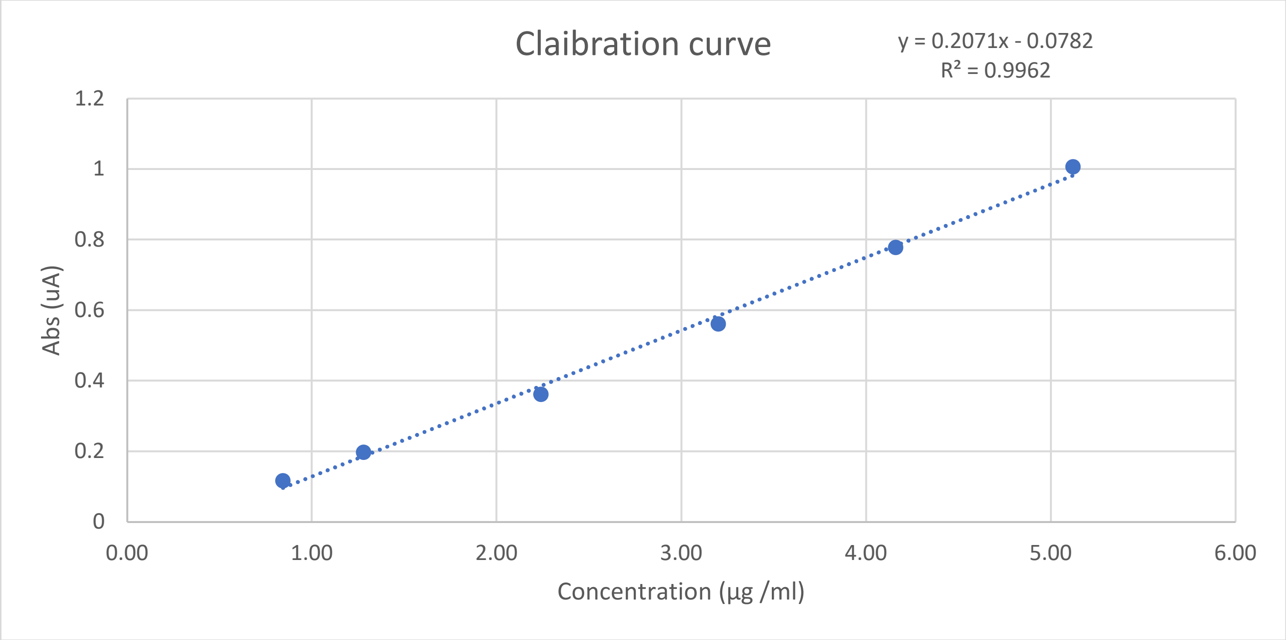

b

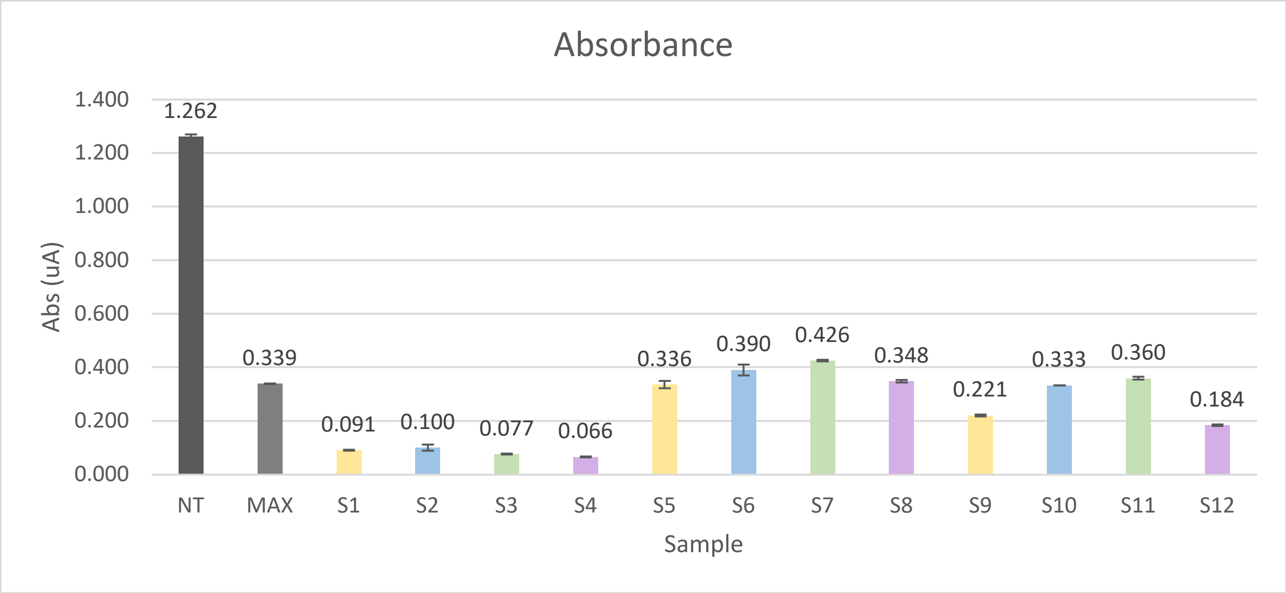

c

Figure S10. (**a**) **SNT:** OMW non treated; **SMAX:** OMW treated with H_2O_2 (10% v/v); **S1:** OMW treated with H_2O_2 (10% v/v) + PhSe)$_2$ (0.6% w/w) at room condition; **S2:** OMW treated with H_2O_2 (10% v/v) + PhSe)$_2$ (0.6% w/w) under BlueLed irradiation; **S3:** OMW treated with H_2O_2 (10% v/v) + PhSe)$_2$ (0.6% w/w) under GreenLed irradiation; **S4:** OMW treated with H_2O_2 (10% v/v) + PhSe)$_2$ (0.6% w/w) under UV irradiation; **S5:** OMW treated with H_2O_2 (1% v/v) + PhSe)$_2$ (0.06% w/w) at room condition; **S6:** OMW treated with H_2O_2 (1% v/v) + PhSe)$_2$ (0.06% w/w) under BlueLed irradiation; **S7:** OMW treated with H_2O_2 (1% v/v) + PhSe)$_2$ (0.06% w/w) under GreenLed irradiation; **S8:** OMW treated with H_2O_2 (1% v/v) + PhSe)$_2$ (0.06% w/w) under UV irradiation; **S9:** OMW treated with H_2O_2 (0.1% v/v) + PhSe)$_2$ (0.006% w/w) at room condition; **S10:** OMW treated with H_2O_2 (0.1% v/v) + PhSe)$_2$ (0.006% w/w) under BlueLed irradiation; **S11:** OMW treated with H_2O_2 (0.1% v/v) + PhSe)$_2$ (0.006% w/w) under GreenLed irradiation; **S12:** OMW treated with H_2O_2 (0.1% v/v) + PhSe)$_2$ (0.006% w/w) under UV irradiation. (**a**) F-C quantification (Materials and Methods, paragraph 3.3.5) of extracted OMW following the procedure reported in Materials and Methods, paragraph 3.3.3. (**b**) Calibration curve of F-C quantification carried out with the procedure reported in Materials and Methods paragraph 3.3.5. (**c**) Absorbance of the sample quantified using the procedure reported in Materials and Methods paragraph 3.3.5; Experiments were performed in triplicates and represented as means. Standard deviations are reported as error bar. All the experiments are statistically significant ($p < 0.05$ vs. NT, p values were calculated applying the unpaired Student's *t* test).

Table S2. Absorbance Means, Standard Deviations and p-value of samples.

**Sample**	Absorbance Means	Standard Deviations	p-Value [a]
NT	1.262	0.00748	-
MAX	0.339	0.000804	0.0001
S1	0.091	0.002193	0.0001
S2	0.100	0.0116	0.0001
S3	0.077	0.0016	0.0001
S4	0.066	0.002166	0.0001
S5	0.336	0.013948	0.0001
S6	0.390	0.020252	0.0001

S7	0.426	0.003151	0.0001
S8	0.348	0.005198	0.0001
S9	0.221	0.003993	0.0001
S10	0.333	0.000266	0.0001
S11	0.360	0.005183	0.0001
S12	0.184	0.002845	0.0001

[a] $p < 0.05$ vs. NT, p values were calculated applying the unpaired Student's *t* test.

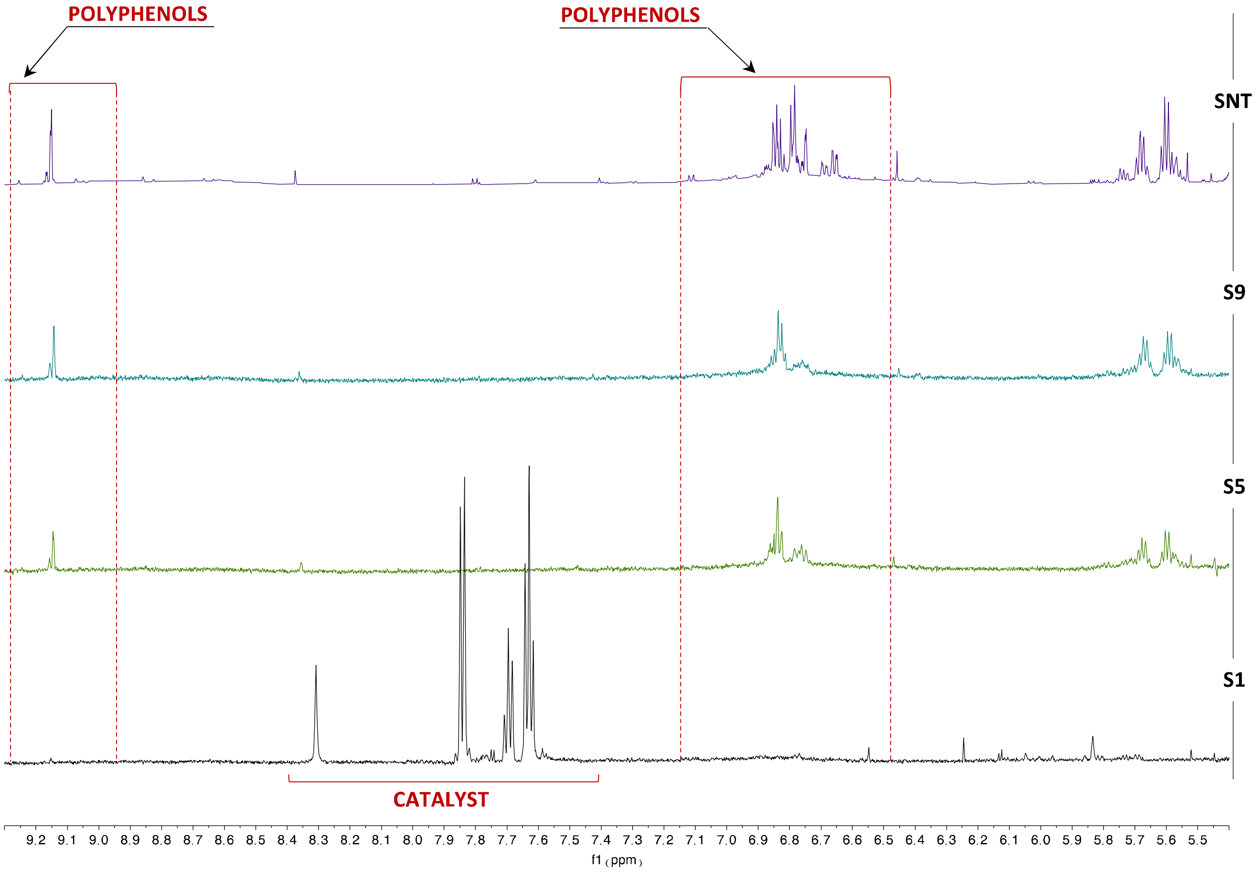

Figure S11. Superimposed spectra of the extracted OMW samples. **SNT:** OMW non treated; **S1:** OMW treated with H_2O_2 (10% v/v) + PhSe)$_2$ (0.6% w/w) at room condition; **S5:** OMW treated with H_2O_2 (1% v/v) + PhSe)$_2$ (0.06% w/w) at room condition; **S9:** OMW treated with H_2O_2 (0.1% v/v) + PhSe)$_2$ (0.006% w/w) at room condition.

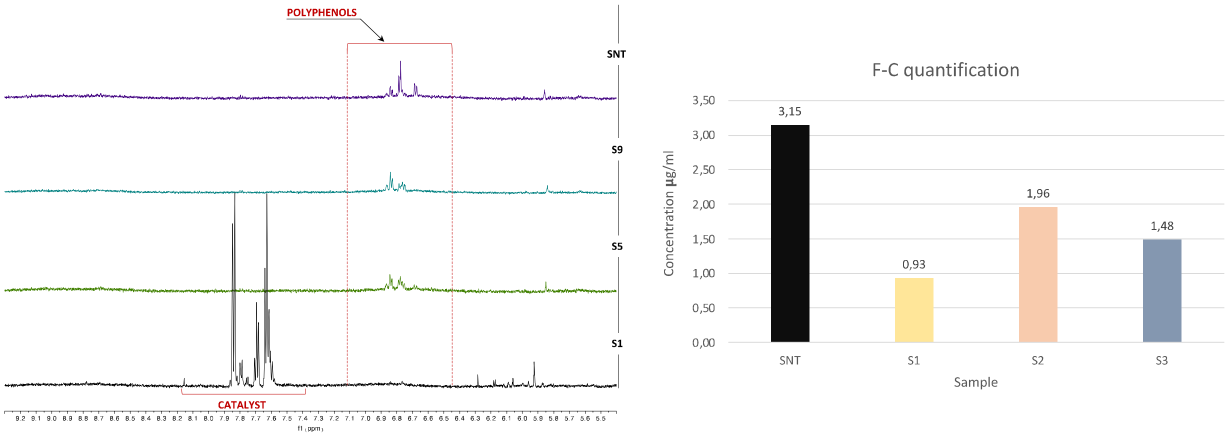

a

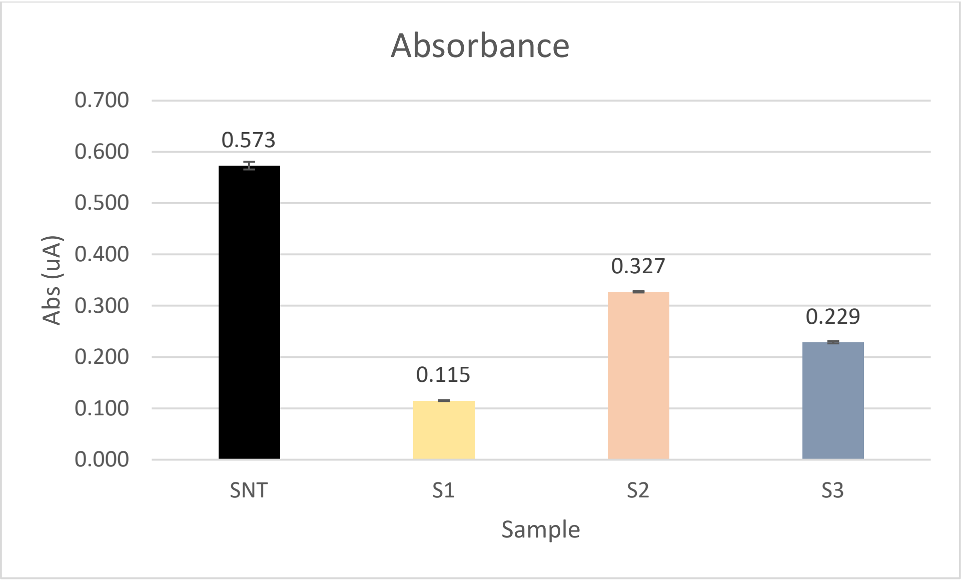

b

Figure S12. (**a**) Quantification of OMW samples extracted under acid conditions. (**b**) Absorbance of the sample quantified using procedure reported in Materials and Methods paragraph 3.3.5.; Experiments were performed in triplicates and represented as means. Standard deviations are reported as error bar. All the experiments are statistically significant ($p < 0.05$ vs. NT, p values were calculated applying the unpaired Student's *t* test). (**a**) Superimposed spectra and F-C quantification of the extracted OMW samples. **SNT:** OMW non treated; **S1:** OMW treated with H_2O_2 (10% v/v) + PhSe)$_2$ (0.6% w/w) at room condition; **S5:** OMW treated with H_2O_2 (1% v/v) + PhSe)$_2$ (0.06% w/w) at room condition; **S9:** OMW treated with H_2O_2 (0.1% v/v) + PhSe)$_2$ (0.006% w/w) at room condition.

Table S3. Absorbance Means, Standard Deviations and p-value of samples.

**Sample**	Absorbance Means	Standard Deviations	p-Value [a]
NT	0.573	0.00759	
S1	0.115	0.000825	0.0001
S2	0.327	0.001208	0.0001
S3	0.229	0.002209	0.0001

[a] $p < 0.05$ vs. NT, p values were calculated applying the unpaired Student's *t* test.

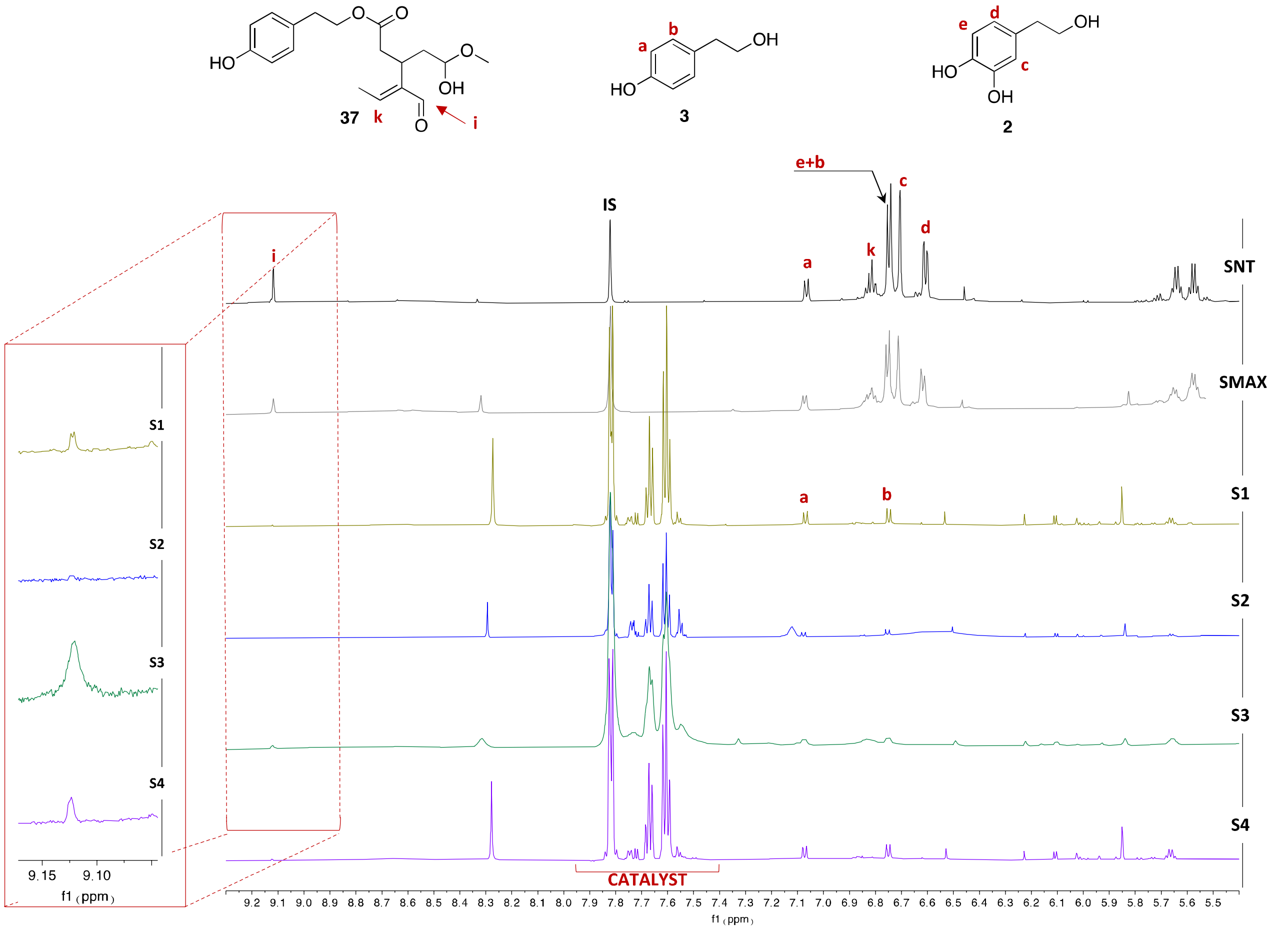

a

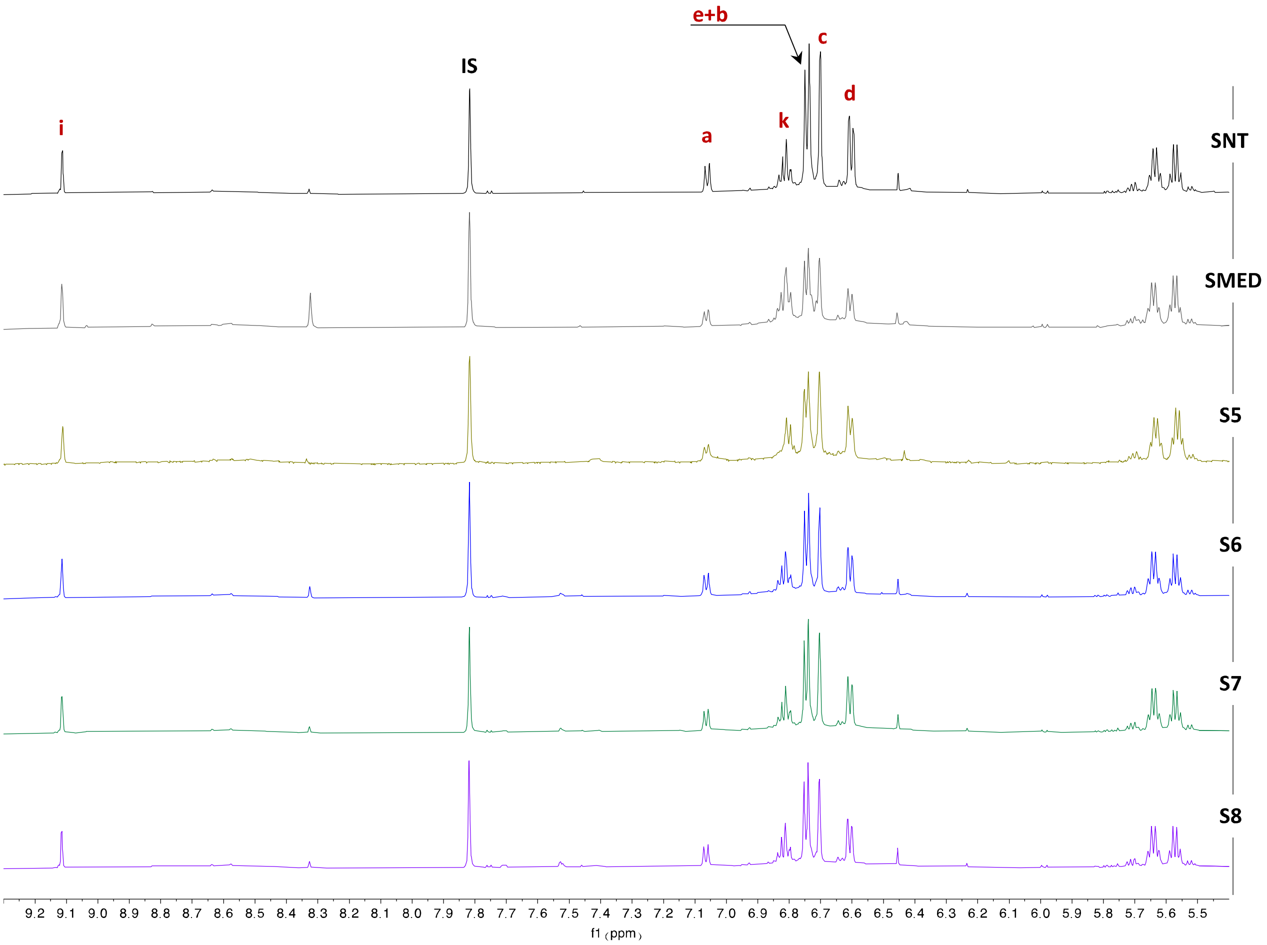

e+b
c
IS
d
i
k
a
SNT
SMED
S5
S6
S7
S8
9.2 9.1 9.0 8.9 8.8 8.7 8.6 8.5 8.4 8.3 8.2 8.1 8.0 7.9 7.8 7.7 7.6 7.5 7.4 7.3 7.2 7.1 7.0 6.9 6.8 6.7 6.6 6.5 6.4 6.3 6.2 6.1 6.0 5.9 5.8 5.7 5.6 5.5
f1 (ppm)

b

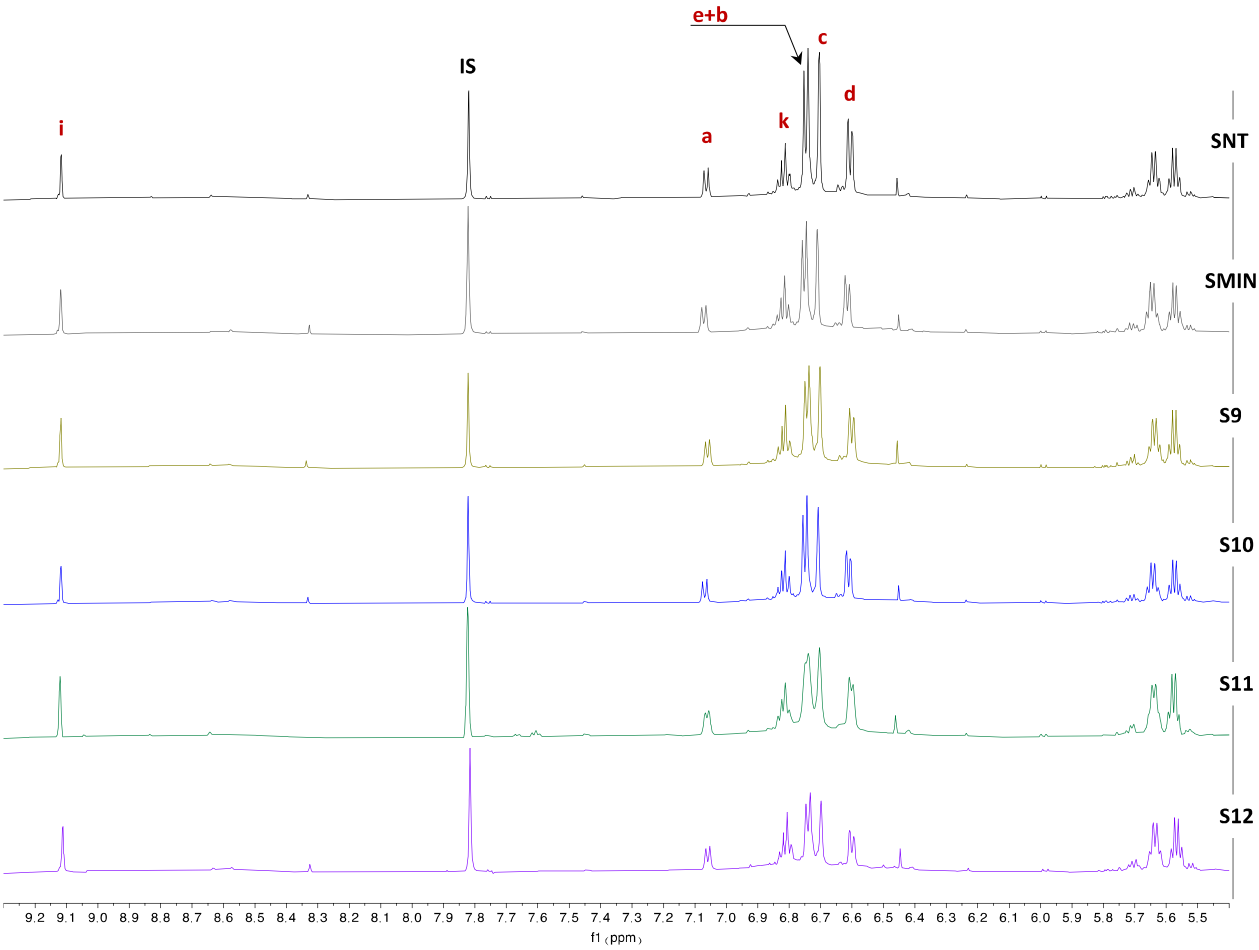

c

Figure S13. (**a**) ^{1}H-NMR quantification of reaction performed applying maximum amount of catalyst and oxidant. **SNT:** OMW non treated; **SMAX:** OMW treated with H_2O_2 (10% v/v); **S1:** OMW treated with H_2O_2 (10% v/v) + PhSe)₂ (0.6% w/w) at room condition; **S2:** OMW treated with H_2O_2 (10% v/v) + PhSe)$_2$ (0.6% w/w) under BlueLed irradiation; **S3:** OMW treated with H_2O_2 (10% v/v) + PhSe)$_2$ (0.6% w/w) under GreenLed irradiation; **S4:** OMW treated with H_2O_2 (10% v/v) + PhSe)$_2$ (0.6% w/w) under UV irradiation; **IS:** internal standard and corresponds to the proton of aldehydic group in DMF, used as internal standard (for the quantification the proton of one of two methyl groups with a signal at 2.98 ppm was used). (**b**) ^{1}H-NMR quantification of reaction performed applying intermediate amount of catalyst and oxidant. **SNT:** OMW non treated; **SMED:** OMW treated with H_2O_2 (1% v/v); **S5:** OMW treated with H_2O_2 (1% v/v) + PhSe)$_2$ (0.06% w/w) at room condition; **S6:** OMW treated with H_2O_2 (1% v/v) + PhSe)$_2$ (0.06% w/w) under BlueLed irradiation; **S7:** OMW treated with H_2O_2 (1% v/v) + PhSe)$_2$ (0.06% w/w) under GreenLed irradiation; **S8:** OMW treated with H_2O_2 (1% v/v) + PhSe)$_2$ (0.06% w/w) under UV irradiation; **IS:** internal standard and corresponds to the proton of aldehydic group in DMF, used as internal standard (for the quantification the proton of one of two methyl groups with a signal at 2.98 ppm was used). (**c**) ^{1}H-NMR quantification of reaction performed applying minimum amount of catalyst and oxidant. **SNT:** OMW non treated; **SMED:** OMW treated with H_2O_2 (0.1% v/v); **S5:** OMW treated with H_2O_2 (0.1% v/v) + PhSe)$_2$ (0.006% w/w) at room condition; **S6:** OMW treated with H_2O_2 (0.1% v/v) + PhSe)$_2$ (0.006% w/w) under BlueLed irradiation; **S7:** OMW treated with H_2O_2 (0.1% v/v) + PhSe)$_2$ (0.006% w/w) under GreenLed irradiation; **S8:** OMW treated with H_2O_2 (0.1% v/v) + PhSe)$_2$ (0.006% w/w) under UV irradiation; **IS:** internal standard and corresponds to the proton of aldehydic group in DMF, used as internal standard (for the quantification the proton of one of two methyl groups with a signal at 2.98 ppm was used).

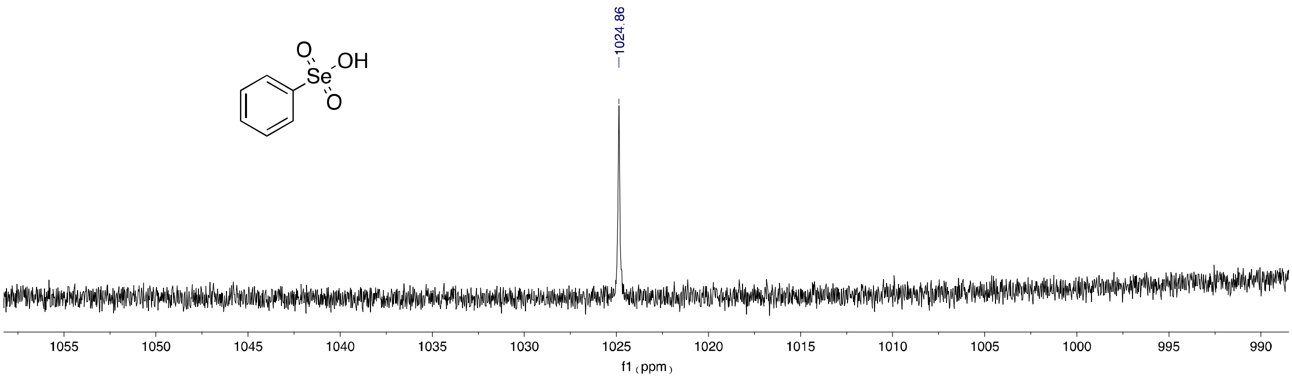

Figure S14. ^{77}Se-NMR spectrum of **1** after oxidation dissolved in D_2O [10].

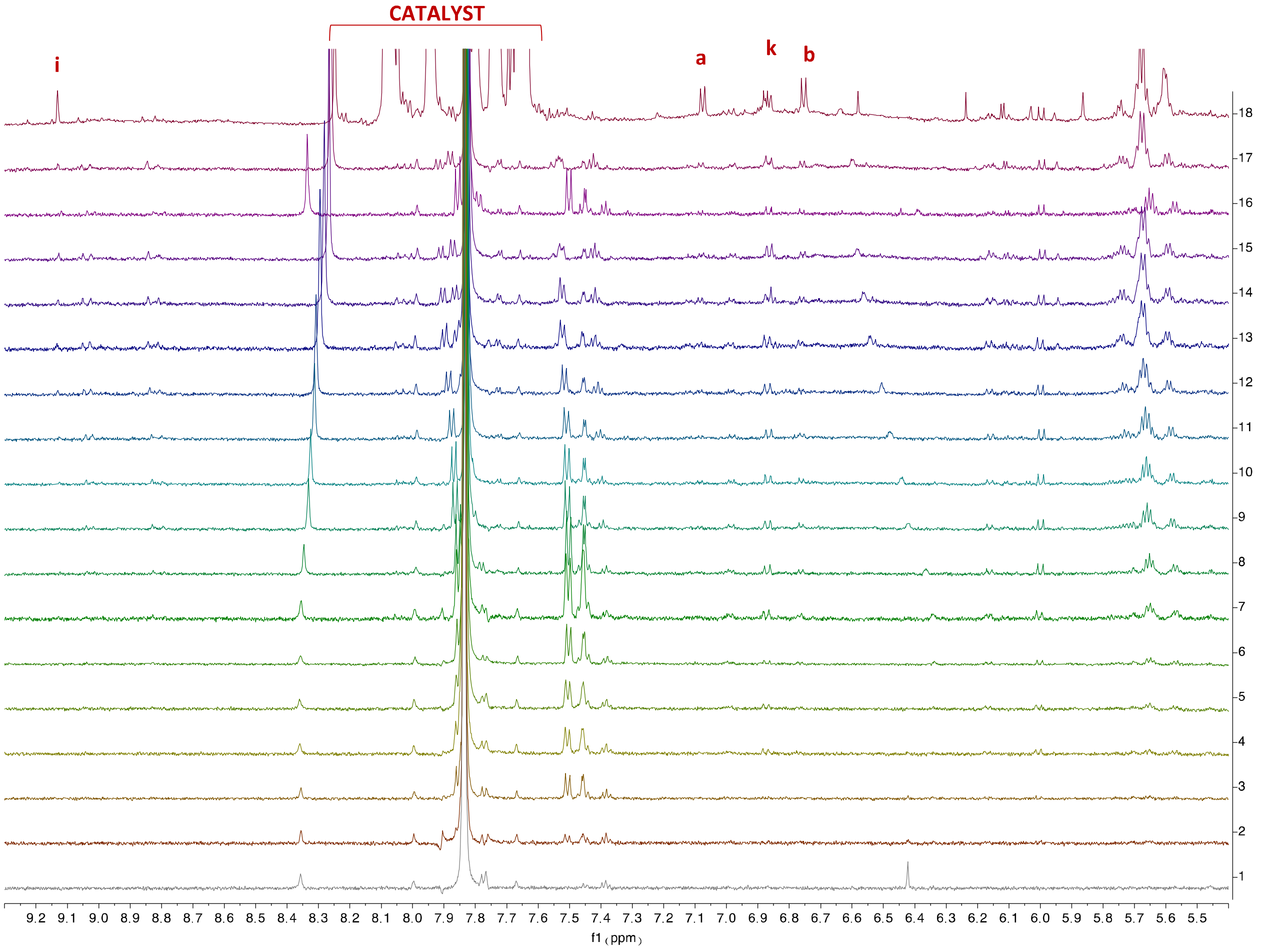

Figure S15. Superimposing of spectra of treated OMW aliquots recovered after column (spectrum 18: OMW before column; spectra 1-17 correspond to aliquot 1 to 17).

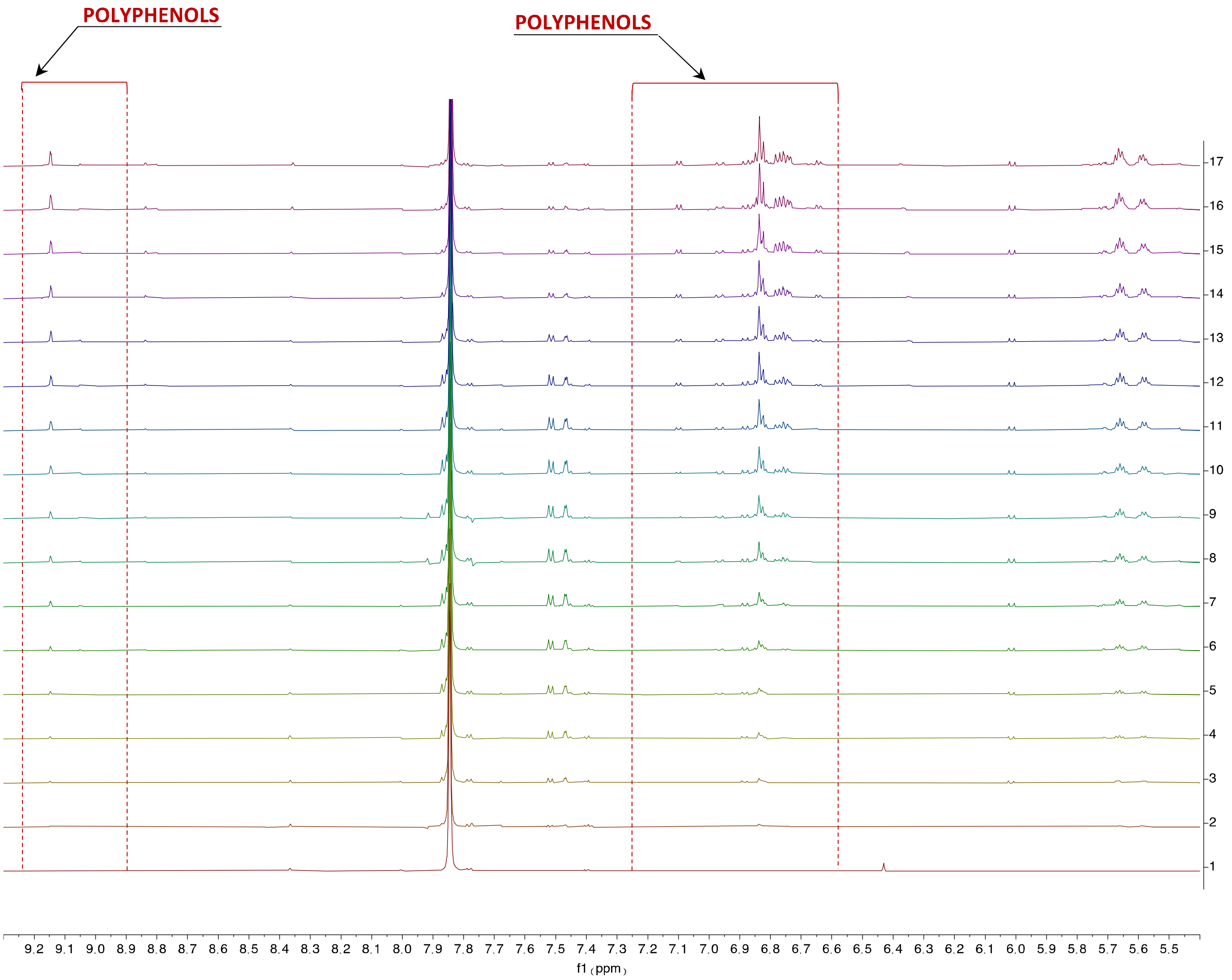

Figure S16. Superimposing of spectra of NON-treated OMW aliquots recovered after column (spectrum 1-17 correspond to aliquot 1 to 17).

References

1. Jastrzebski, R.; Weckhuysen, B.M.; Bruijnincx, P.C.A. Catalytic Oxidative Cleavage of Catechol by a Non-Heme Iron(Iii) Complex as a Green Route to Dimethyl Adipate. *Chem. Commun.* **2013**, *49*, 6912, doi:10.1039/c3cc42423e.
2. Grill, J.M.; Ogle, J.W.; Miller, S.A. An Efficient and Practical System for the Catalytic Oxidation of Alcohols, Aldehydes, and α,β-Unsaturated Carboxylic Acids. *J. Org. Chem.* **2006**, *71*, 9291–9296, doi:10.1021/jo0612574.
3. Kastelic, J.; Hodnik, Ž.; Šket, P.; Plavec, J.; Lah, N.; Leban, I.; Pajk, M.; Planinšek, O.; Kikelj, D. Fluconazole Cocrystals with Dicarboxylic Acids. *Cryst. Growth Des.* **2010**, *10*, 4943–4953, doi:10.1021/cg1010117.
4. Jones, I.C.; Sharman, G.J.; Pidgeon, J. 1H and13C NMR Data to Aid the Identification and Quantification of Residual Solvents by NMR Spectroscopy. *Magn. Reson. Chem.* **2005**, *43*, 497–509, doi:10.1002/mrc.1578.
5. Saikia, B.; Borah, P. A New Avenue to the Dakin Reaction in H_2 O_2 –WERSA. *RSC Adv.* **2015**, *5*, 105583–105586, doi:10.1039/C5RA20133K.
6. Stern, A.J.; Swenton, J.S. Addition of Organolithium Reagents to Quinone Silyl Methyl Monoketals. A Useful Expedient in the Synthesis of p-Quinols Having Acid-Sensitive Groups. *J. Org. Chem.* **1988**, *53*, 2465–2468, doi:10.1021/jo00246a013.
7. Giurg, M.; Kowal, E.; Muchalski, H.; Syper, L.; Młochowski, J. Catalytic Oxidative Domino Degradation of Alkyl Phenols Towards 2- and 3-Substituted Muconolactones. *Synth. Commun.* **2008**, *39*, 251–266, doi:10.1080/00397910802369687.
8. Krasowska, D.; Begini, F.; Santi, C.; Mangiavacchi, F.; Drabowicz, J.; Sancineto, L. Ultrasound-Assisted Synthesis of Alkali Metals Diselenides (M2Se2) and Their Application for the Gram-Scale Preparation of 2,2′-Diselenobis(Benzoic Acid). *Arkivoc* **2019**, *2019*, 24.
9. Sysolyatin, S.V.; Kryukov, Y.A.; Malykhin, V.V.; Muradov, K. K.; Chernysheva, G. A.; Aliev, O. I.; Smol'yakova, V. I.; Anishchenko, A. M.; Sidekhmenova, A. V.; Shamanaev, A. Y.; Plotnikov, M. B. p-Tyrosol: a new synthetic method and new types of pharmacological activity. *Russ Chem Bull* **2015**, *64*, 2210–2214, doi.org/10.1007/s11172-015-1140-y.
10. Sands, K.N.; Mendoza Rengifo, E.; George, G.N.; Pickering, I.J.; Gelfand, B.S.; Back, T.G. The Unexpected Role of Se ^{VI} Species in Epoxidations with Benzeneseleninic Acid and Hydrogen Peroxide. *Angew. Chem. Int. Ed.* **2020**, *59*, 4283–4287, doi:10.1002/anie.201913566.
